# Supplementary material for: Impact of Negative Feedbacks on De Novo Pyrimidines Biosynthesis in Escherichia coli
Source: Int J Mol Sci. 2023 Mar 2;24(5):4806. doi: 10.3390/ijms24054806 (PMC10003070; doi:10.3390/ijms24054806)
Supplement: Supplementary file 1 [file ijms-24-04806-s001.zip › SFs_legends_Akberdin_etal_2023.pdf]

Figure SF1.
Boxplots for each parameter is plotted on a separate panel named after that parameter. Boxplots for parameters with statistically significant differences according to Welch test (p-value < 0.005) are showed on the page 2.
mog: parameters fitted for Bennet dataset.
mug: parameters fitted for Ishi dataset.

Figures SF2-SF4.
System dynamics regime for different combinations of parameters. Each point in 3D plots plot represents one analyzed combination of values. Green and red colors correspond to stable and oscillatory regimes, respectively.
p5: h_{ump1} - Hill coefficient for nonlinear impact of UMP to the activity of carbamoylphosphate synthetase (regulatory loop 1).
p7: h_{udp1} - Hill coefficient for nonlinear impact of UDP to the activity of carbamoylphosphate synthetase
p9: h_{utp1} - Hill coefficient for nonlinear impact of UTP to the activity of carbamoylphosphate synthetase
p35: r – designates the impact of non-competitive mechanism of UTP inhibition to the regulation of UMP phosphorylation (regulatory loop 6).

Table SF5
Sensitivity analysis results. Each cell in the table represents a scaled sensitivity coefficient for a certain variable to the change of the kinetic parameter value. A positive/negative value of the measure indicates that a certain variable will increase/decrease with the increase of a parameter value.
