# Supplementary material for: Impact of Negative Feedbacks on De Novo Pyrimidines Biosynthesis in Escherichia coli
Source: Int J Mol Sci. 2023 Mar 2;24(5):4806. doi: 10.3390/ijms24054806 (PMC10003070; doi:10.3390/ijms24054806)
Supplement: Supplementary file 1 [file ijms-24-04806-s001.zip › SupplTexts_Akberdin_etal_2023.pdf]

Supplementary Text S1.

Description of models of elementary subsystems of pyrimidine biosynthesis in *E. coli* cell.

The Michaelis-Menten equations were used to describe the rates of the enzymatic reactions V_3-V_6, V_8: $V = \frac{\mathrm{k_{cat}} \cdot E \cdot S}{\mathrm{Km} + S}$. Their parameters were taken from the literature (see Table S1). It is worth noting that data on the reversibility and experimentally established ping-pong mechanism of the enzymatic reaction [Björnberg et al., 1999] were used to describe the V_4 rate. To describe the V_9 rate, a formula was used that was obtained after refining a previously published mathematical model [MacDonell et al., 2004]. For rates V_1, V_2, V_7, original models were developed in terms of generalized Hill functions [Likhoshvai and Ratushny, 2007], which reproduce known experimental data for each enzymatic reaction. The rates V_{10},V_{11} and V_{22},V_{23}, which characterize the processes of the outflow of synthesized pyrimidines for the formation of RNA and DNA molecules, as well as their degradation, are proportional to the constants k_9, k_{10} and k_{22}, k_{23}, respectively, whose values are presented in Table S1.

Below is a description of the functions for rates V_1, V_2, V_7.

V_1 – ***the synthesis rate of carbamoyl phosphate, a precursor of pyrimidine nucleotides***

A key step in the biosynthesis of pyrimidine nucleotides is the synthesis of carbamoyl phosphate from ATP, glutamine, and bicarbonate. The reaction is catalyzed by the enzyme carbamoyl-phosphate synthetase (EC 6.3.5.5). Allosteric regulators of enzyme activity are inosine monophosphate (IMP), ornithine (ORN), and uridine monophosphate (UMP) [Robin et al., 1989; Pierrat et al., 2002; Eroglu, 2002]. Uridine diphosphate (UDP) and uridine triphosphate (UTP) regulate the reaction by an unspecified mechanism [Anderson et al., 1966; Eroglu, 2002].

$$V_1 = \mathrm{k_{cat1}} \cdot E1 \cdot fa \cdot fb \cdot fr,$$

$$fa = \frac{1 + \delta\mathrm{ORN} \cdot \left(\frac{ORN}{\mathrm{KORN1}}\right)^{\mathrm{hORN1}}}{1 + \left(\frac{ORN}{\mathrm{KORN1}}\right)^{\mathrm{hORN1}}},\quad fb = \frac{\frac{BC}{\mathrm{KmBC}} \cdot \frac{GLN}{\mathrm{KmGLN}} \cdot \left(\frac{ATP}{\mathrm{KATP0.5}}\right)^2}{\left(1 + \frac{BC}{\mathrm{KmBC}}\right) \cdot \left(1 + \frac{GLN}{\mathrm{KmGLN}}\right) \cdot \left(1 + \frac{ATP}{\mathrm{KATP0.5}}\right)^2}$$

$$fr = \frac{1 + \delta\mathrm{IMP} \cdot \left(\frac{IMP}{\mathrm{KIMP1}}\right)}{1 + \left(\frac{UMP}{\mathrm{KUMP1}}\right)^{\mathrm{hUMP1}} + \left(\frac{IMP}{\mathrm{KIMP1}}\right) + \left(\frac{UDP}{\mathrm{KUDP1}}\right)^{\mathrm{hUDP1}} + \left(\frac{UTP}{\mathrm{KUTP1}}\right)^{\mathrm{hUTP1}}}, \tag{1}$$

$$sCAP = 1, \quad sE1, sORN, sBC, sGLN, sATP, sIMP, sUDP, sUTP = 0$$

Here $E1$ is the concentration of carbamoyl-phosphate synthetase; $\mathrm{k_{cat1}}$ is the catalytic turnover constant of carbamoyl-phosphate synthetase; *CMP*, *BC, GLN, ATP, ORN, IMP, UMP, UDP*, and *UTP* are concentrations of carbamoyl phosphate, bicarbonate, glutamine, ATP, ornithine, IMP, UMP, UDP, and UTP, respectively; $sCMP, sE1, sORN, sBC, sGLN, sATP, sIMP, sUDP, sUTP$ - stoichiometric coefficients, $\mathrm{KATP0.5}$ - threshold constant of the effectiveness of ATP effect on the reaction rate; KmBC, KmGLN - Michaelis constants for bicarbonate and glutamine, respectively; KUMP1, KUDP1, KUTP1 - enzyme inhibition constants by UMP, UDP, and UTP, respectively; KORN1 and KIMP1 - enzyme activation constants by ornithine and IMP, respectively; $\delta\mathrm{IMP}, \delta\mathrm{ORN}$ - constants characterizing the specific activity of the enzyme under the action of IMP and ornithine, respectively; hUMP1, hUDP1, hUTP1 and hORN1 - constants

characterizing the nonlinearity degree of the influence of UMP, UDP, UTP and ornithine, respectively, on the activity of the enzyme.

The synthesis rate of carbamoyl phosphate V_1 is described by function (1). The multipliers fa, fb, and fr reflect the assumption of independence of the interaction processes of substrates with the enzyme, the action of ornithine, and the effect of regulatory molecules UMP, UDP, UTP, and IMP.

In turn, the function fb describes the independent interaction of substrates with specific sites of the enzyme. Hyperbolic Michaelis-Menten kinetics is observed for the bicarbonate and glutamine substrates, and quadratic-hyperbolic for ATP (Figs. S1A and S1B). The values of the constants $KmBC$, $KmGLN$, and $KATP0.5$ were taken from [Robin et al.,1989].

The function fr describes the processes of inhibition of the enzyme activity by UMP, UDP and UTP substances and its activation by IMP substance. The type of function reflects the assumption of the competitive nature of the action of these substances on the enzyme activity [Boettcher and Meister, 1982; Wild et al., 1989]. This assumption was confirmed for IMP and UMP by mutation research [Fresquet et al., 2000] and X-ray diffraction analysis [Thoden et al., 2004]. On this basis, the numerator of the function fr contains a term depending on the variable corresponding to the activator concentration (IMP), while the denominator contains terms depending on the variables corresponding to the concentrations of all the reaction regulators (UMP, UDP, UTP, and IMP). The values of the constants included in this function and characterizing the action of UMP and IMP were taken from the experimental studies or selected from the experimental data presented in Fig. S1C [Robin et al.,1989]. The constants for UDP, UTP were estimated from the data given in [Anderson et al., 1966].

The function fa describes the process of enzyme activation by ornithine. Ornithine reduces the effect of UMP inhibition and functions independently of nucleotide group regulators [Wild et al., 1989]. Additionally, the independence of the regulation of the enzyme activity by ornithine was confirmed by X-ray analysis, which showed that the ornithine binding site does not sterically overlap with the sites of other regulators [Holden et al., 1999].

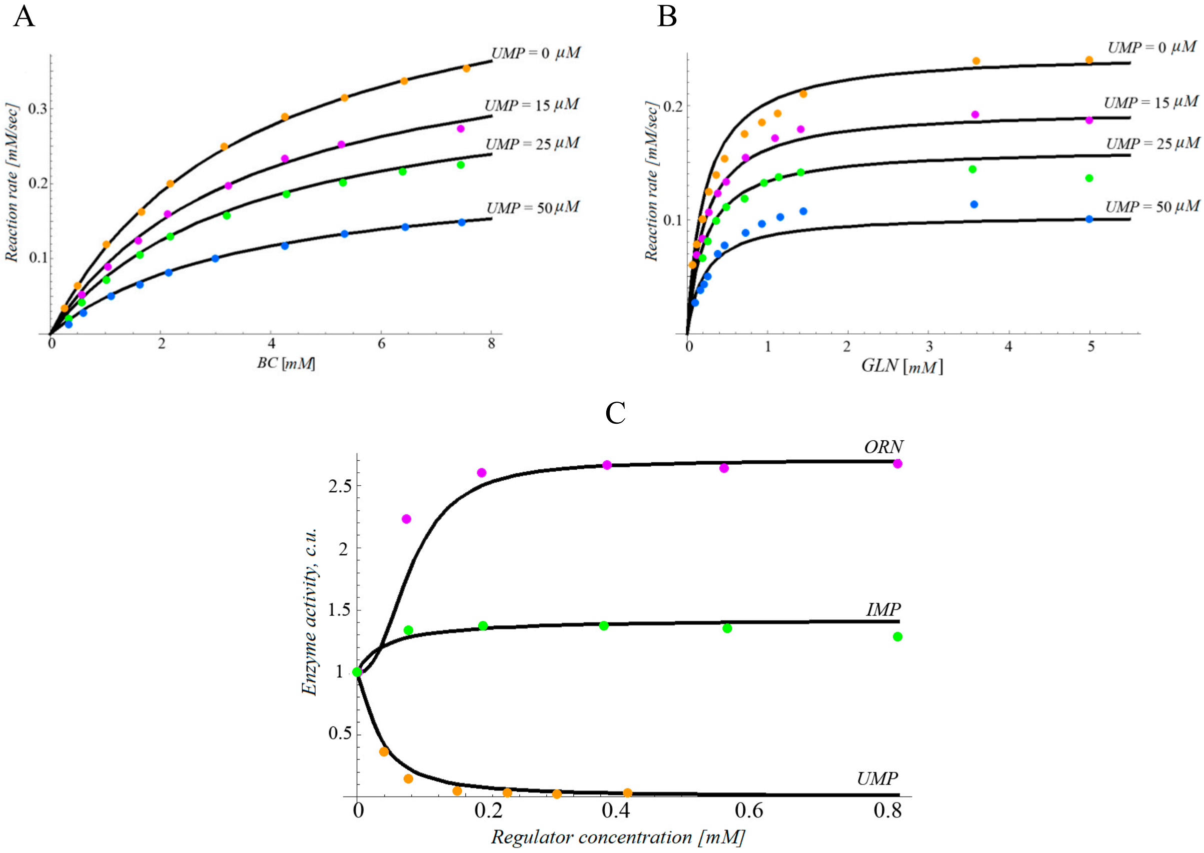

Figure S1. The results of the model (1) adaptation to the experimental data from the article [Robin et al.,1989]. Figures S1A and S1B show the effect of UMP on the rate of the enzymatic reaction catalyzed by carbamoyl-phosphate synthetase as a function of the bicarbonate (A) and glutamine (B) concentration. *ATP* = 20 mM (A, B); *GLN* = 12.5 mM (A); *BC* = 10 mM (B). Figure S1C demonstrates the effect of IMP, UMP, and ornithine on enzyme activity. Dots are experimental values, curves are calculated by the model (1).

Model (1) is an extension of the model proposed by Rodriguez et al. in 2005 [Rodriguez et al., 2005]. Model (1) enables to calculate the rate of the enzymatic reaction catalyzed by carbamoyl-phosphate synthetase, taking into account the concentrations of three substrates and five regulators, whereas Rodriguez model considers two substrates and one regulator. In addition, model (1) takes into account the experimental data on the nonlinear nature of the kinetics of the UMP effect on the reaction rate [Robin et al.,1989], which is expressed in formula (1) as the presence of the Hill coefficient hUMP1 equal to 1.4, while the kinetics of the effect of UMP on the rate is assumed linear in inverse coordinates in the Rodrigues model. Thus, the developed model (1) incorporates the mechanisms of enzyme functioning that were not taken into account in the Rodriguez model that provides a more accurate description of the carbamoyl-phosphate synthetase enzyme kinetics.

***V_2* – the rate of the first specific reaction of pyrimidine nucleotides biosynthesis – carbamoyl aspartate formation**

The first specific reaction in the biosynthesis of pyrimidine nucleotides is the formation of carbamoyl aspartate from carbamoyl phosphate and aspartate, which is catalyzed by aspartate transcarbamoylase (*EC 2.1.3.2*). The allosteric regulators of aspartate transcarbamoylase are ATP, CTP, and UTP. They interact with the same binding sites on the regulatory subunits of the enzyme, but their action is differently directed. Enzyme binding to ATP increases enzyme activity, whereas binding to CTP and/or UTP promotes a decrease in the activity [Wild et al., 1989, Wales et al., 1999]. It should only be noted that the effect of UTP is much weaker than that of CTP, but in the presence of CTP its effect on the enzyme activity is much more pronounced [Wild et al., 1989, Wales et al., 1999].

In model (2), the rate of carbamoyl aspartate synthesis is described taking into account the concentrations of substrates, aspartate and carbamoyl phosphate, and the regulators ATP, CTP, and UTP.

$$V_2 = kcat2 \cdot E2 \cdot fa \cdot fb \cdot fc,\ fa = \frac{(xASP)^{hASP21}}{1+(xASP)^{hASP22}},\ xASP = \frac{ASP}{S0.5ASP2},\ fb = \frac{(xCAP)^{hCAP}}{1+(xCAP)^{hCAP}},\ xCAP = \frac{CAP}{S_{0.5CAP2}},$$

$$fc = \frac{1+\delta_{CTP}\cdot\left(\frac{CTP}{kCTP}\right)+\delta_{ATP}\cdot\left(\frac{ATP}{kATP}\right)+\delta_{UTP}\cdot\left(\frac{UTP}{kUTP}\right)}{1+\left(\frac{CTP}{kCTP}\right)+\left(\frac{ATP}{kATP}\right)+\left(\frac{UTP}{kUTP}\right)+\omega_{CTP,UTP}\cdot\left(\frac{CTP}{kCTP}\right)\left(\frac{UTP}{kUTP}\right)}, \qquad (2)$$

$$sCAP = -1, sCAAP = 1, sE2, sASP, sCTP, sATP, sUTP = 0,$$

Here, $E2$ is the aspartate-transcarbamoylase concentration; k_{cat2} is the catalytic constant of the enzyme turnover; *ASP, CAP, ATP, UTP, CTP* are the concentrations of aspartate, carbamoyl phosphate, ATP, UTP, and CTP, respectively; $S_{0.5ASP2}$,, $S_{0.5CAP2}$ – constants characterizing ASP and CAP concentration at half maximum rate; *kCTP*, *kUTP* - constants of CTP and UTP enzyme inhibition, respectively; *kATP* - constant of ATP enzyme activation; δ_{CTP}, δ_{ATP}, δ_{UTP}– constants characterizing the specific activity of the enzyme under the action of CTP, ATP and UTP, respectively; *hCAP* and *hASP*21 *(hASP*22*)* – constants characterizing the degree of nonlinearity of the influence of CAP and ASP, respectively, on the enzyme activity; $\omega_{CTP,UTP}$ – a constant characterizing the degree of mutual effect of CTP and UTP influence.

The reaction rate in model (2) is described by the product of three functions. The functions fa and fb describe the independent interaction of aspartate and carbamoyl phosphate with specific enzyme sites. However, the interaction does not follow the classical Michaelis-Menten mechanism, since the saturation curves for them have a complex sigmoidal form, as reflected in Figures S2A and S2B, plotted according to data from [Xu et al.,1991]. The constants characterizing the concentrations of aspartate and carbamoyl phosphate at which ½ Vmax of the reaction rate is realized were taken from the same work [Xu et al.,1991].

The function fc describes the inhibition of the enzyme activity by UTP and CTP and its activation by ATP (see Fig. S2C). The type of function reflects the assumption of the competitive nature of the action of ATP and CTP on the enzyme activity [Changeux et al., 1968] and takes into account the synergistic action of UTP and CTP [Wild et al., 1989; Wales et al., 1999]. The presence of variables corresponding to the concentrations of ATP, UTP, and CTP in the numerator of this function is explained by the presence of an activation effect in the presence of ATP and incomplete inhibition of the enzyme activity in the presence of UTP or CTP.

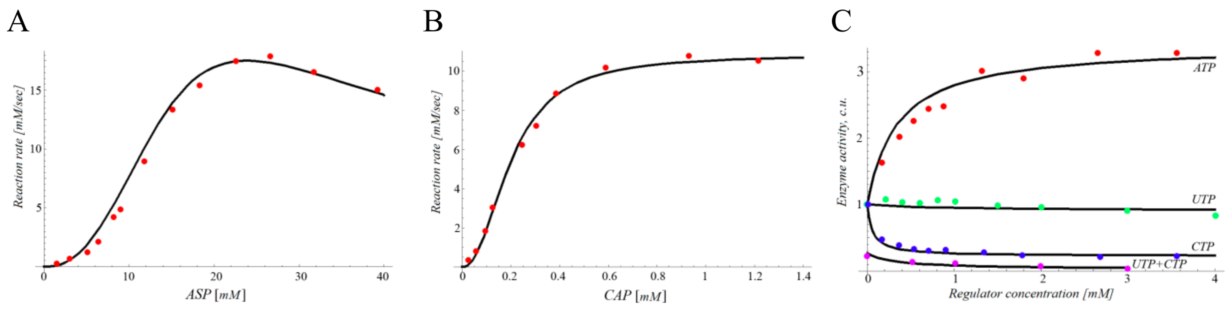

Figure S2. The results of the model (2) adaptation to the experimental data from Xu et al. [1991] (A, B) and Wales et al. [1999] (B). Figures S2A and S2B show the dependence of the reaction rate catalyzed by aspartate transcarbamoylase on the concentration of aspartate (S2A) and carbamoyl phosphate (S2B). *CAP* = 4.8 mM (A); *ASP* = 30 (B). Figure S2C shows the effect of ATP, UTP, CTP, and CTP+UTP on enzyme activity. The dots are experimental values; the curves are calculations according to the model (2).

It can be seen from the view of fc function that the effect of the regulatory molecules ATP, UTP, and CTP on the activity of the enzyme (see Fig. S2C) is well described by a rational function containing single Hill coefficients, although the enzyme contains six regulatory subunits in the form of three dimeric molecules. That is, the kinetics describing the enzyme activity depending on the regulatory molecules has a simpler form than expected based on the structure of the enzyme.

V_7 – *the reaction rate of UMP phosphorylation – the first pyrimidine nucleotide*

The phosphorylation reaction of the first pyrimidine nucleotide of uridine monophosphate (UMP) is catalyzed by uridine monophosphate kinase (*EC 2.7.4.22*, *pyrH* gene). The regulators of this enzymatic reaction are GTP (activator) and UTP (inhibitor) [Serina et al., 1995; Briozzo et al., 2005], whose binding sites on the enzyme do not overlap [Meyer et al., 2008; Marco-Marín et al., 2009].

The model is represented by the formula:

$$V_7 = k_{cat7} \cdot E7 \cdot fa \cdot fb \cdot fc\ ,\ \ fa = \frac{ATP}{KmATP + ATP}\ ,\ \ fb = \frac{1 + \delta_{GTP} \cdot (xGTP)^{hGTP7}}{1 + (xGTP)^{hGTP7}}, xGTP = \frac{GTP}{kGTP7}, \tag{3}$$

$$fc = \frac{UMP}{KmUMP \cdot \left(1 + (1-r) \cdot \left(\frac{UTP}{kUTP71}\right)^{hUTP7}\right) + UMP} \cdot \frac{1}{1 + r \cdot \left(\frac{UTP}{kUTP72}\right)^{hUTP7}},$$

$$sUMP = -1,\ sUDP = +1, sE7, sATP, sGTP, sUTP = 0.$$

Here, $E7$ is the concentration of the enzyme uridine monophosphate kinase; k_{cat7} is the catalytic constant of the enzyme turnover; *UTP, GTP, ATP, UMP* are the concentrations of UTP, GTP, ATP, and UMP, respectively; *KmUMP* and *KmATP* – the Michaelis constant for uridine monophosphate and adenosine triphosphate, respectively; *kUTP71* and *kUTP72* – inhibition

constants of UTP enzyme activity; *kGTP7* – activation constant of GTP enzyme; δ_{GTP} – the specific activity of the enzyme under the action of GTP; *hUTP7* and *hGTP7* – constants characterizing the degree of nonlinearity of the effect of UTP and GTP, respectively, on the enzyme activity; *r* – a constant characterizing the contribution of noncompetitive inhibition to the enzyme regulation, where *r* takes values from 0 to 1; (1-*r*) – a constant characterizing the contribution of competitive inhibition to the enzyme regulation.

The reaction regulation in model (3) is described by the product of three functions: *fa*, *fb*, and *fc*. The function *fa* has the simplest form, which corresponds to the classical Michaelis-Menten mechanism, by which ATP interacts with a specific site of the enzyme. The function *fb* describes the positive kinetics of the effect of GTP on enzyme activity (see Fig. S3B). The function *fc* at a fixed concentration of UTP also describes the UMP interaction by the Michaelis-Menten mechanism. The inhibition of UTP is described by introducing additional factors into the formula: $\left(1+(1-r)\cdot\left(\frac{UTP}{kUTP71}\right)^{hUTP7}\right)$, describing competitive inhibition of the enzyme activity, so this expression is multiplied by the Michaelis constant, and $\frac{1}{1+r\cdot\left(\frac{UTP}{kUTP72}\right)^{hUTP7}}$ describes noncompetitive inhibition. The nonlinear kinetics of UTP inhibition (see Fig. S3A), is accounted for in these summands by the presence of the nonunique Hill coefficient *hUTP*7. The parameters *r* and 1-*r* characterize the contribution of the noncompetitive and competitive inhibition mechanisms to the regulation of the enzyme.

A B

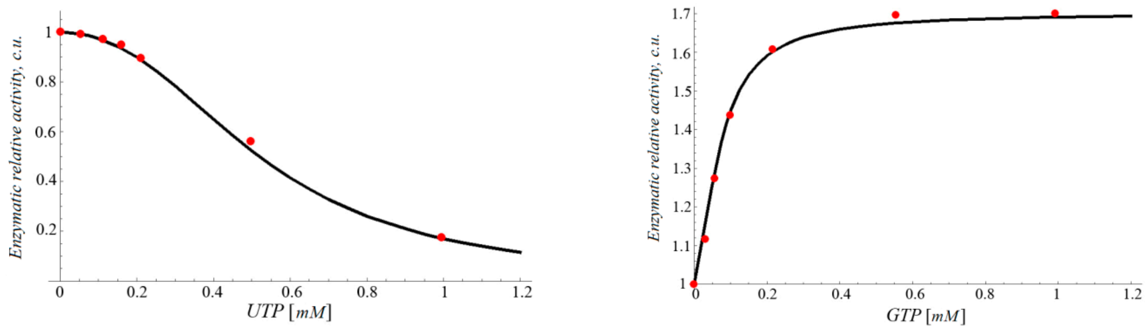

Figure S3. Dependence of uridine monophosphate kinase enzyme activity on the concentration of UTP (A) and GTP (B). The dots correspond to the experimental data from the article [Serina et al., 1995], curves - model (3) calculation. *UMP* = *ATP* = 1 mM for S3A and S3B.

The mathematical model (3) was developed was developed based on data from Serin and coathours [Serina et al., 1995]. They showed that UTP is an allosteric inhibitor of uridine monophosphate kinase activity, but Brizzo and colleagues [Briozzo et al., 2005] based on X-ray analysis of the enzyme demonstrated later that UTP and UMP have overlapping (virtually identical) sites on the enzyme, that is, UTP is a competitive inhibitor, sterically preventing interaction between the UMP substrate and the enzyme. However, given the complex hexameric structure of the enzyme's active form, the presence of allosteric regulation of UMP kinase activity

by UTP cannot be denied. Therefore, both regulatory mechanisms of UMP kinase activity by UTP were taken into account in the model of nucleotide biosynthesis.

Table S1. Parameter values of the pyrimidine nucleotide biosynthesis model (1 in the maintext) adapted to the experimental data. K_{mi} is the Michaelis constant; K_i is a constant characterizing the efficiency of inhibition/activation of the corresponding compounds; h_i is a constant characterizing the degree of nonlinearity of the effect of the corresponding compounds; ω_1 is a constant of the efficiency of the joint effect of substances; δ_i is a constant characterizing the specific activity of the enzyme under the action of the corresponding compounds; r is the constant characterizing the contribution of noncompetitive inhibition to the enzyme regulation, where r takes values from 0 to 1; (1-r) is the constant characterizing the contribution of competitive inhibition to the enzyme regulation; $k0_i$ – baseline activity of the enzyme; *BC, GLN, ATP, ORN, IMP, ASP, Q, OH2, PRPP, GTP* – equilibrium concentrations of metabolites.

Model parameter	Value	Reference	Model parameter	Value	Reference
k_{1f}	12 sec^{-1}	[Pierrat et al., 2002]	QH2	0.09 mM	[Shestopalov et al., 1997, Bekker et al., 2007]
BC	10 mM	[Robin et al., 1989]	$K_{m_{QH2_4}}$	0.01 mM	**
$K_{m_{BC}}$	3.6 mM	[Robin et al., 1989]	K_{OROA_4}	0.0134 mM	[Björnberg et al., 1999]
GLN	3.8 mM	[Bennett et al., 2009]	$K_{m_{OROA_4}}$	0.01 mM	**
$K_{m_{GLN}}$	0.22	[Robin et al., 1989]	k_{5f}	12 sec^{-1}	[Shimosaka et al., 1985]
ATP	9.6 mM	[Bennett et al., 2009]	PRPP	0.26 mM	[Bennett et al., 2009]
$K_{m_{ATP_1}}$	8 mM	[Robin et al., 1989]	$K_{m_{prpp5}}$	0.04 mM	[Shimosaka et al., 1985]
δ_{orn}	3.4	[Pierrat et al., 2002]	$K_{m_{OROA_5}}$	0.03 mM	[Shimosaka et al., 1985]
ORN	0.01 mM	[Bennett et al., 2009]	k_{6f}	198 sec^{-1}	[Donovan&Kushner, 1983]
k_{orn_1}	0.1 mM	[Robin et al., 1989]	$K_{m_{OMP_6}}$	0.006 mM	[Donovan&Kushner, 1983]
horn	2.4	[Robin et al., 1989]*	k_{7f}	332 sec^{-1}	[Serina et al., 1996]
IMP	0.27 mM	[Bennett et al., 2009]	$K_{m_{UMP_7}}$	0.05 mM	[Serina et al., 1995]
δ_{IMP}	1.43	[Robin et al., 1989]*	h_{UTP_7}	2.4	[Serina et al., 1995]*
k_{IMP_1}	0.05 mM	[Robin et al., 1989]	$K_{UTP_{71}}$	0.54 mM	[Serina et al., 1995]*
K_{UMP_1}	0.04 mM	[Robin et al., 1989]	$K_{UTP_{72}}$	0.15 mM	[Serina et al., 1995]*
h_{UMP_1}	1.4	[Robin et al., 1989]*	K_{GTP_7}	0.07 mM	[Serina et al., 1995]*
K_{UDP_1}	0.73 mM	[Anderson&Meister, 1966]*	h_{GTP_7}	1.6	[Serina et al., 1995]*
h_{UDP_1}	1.4	[Anderson&Meister, 1966]*	δ_{GTP_7}	1.71	[Serina et al., 1995]*

K_{UTP_1}	1.03 mM	[Anderson&Meister, 1966]*	r	1	[Serina et al., 1995]
h_{UTP_1}	1.4	[Anderson&Meister, 1966]*		0	[Briozzo et al., 2005]
k_{2f}	1667 sec^{-1}	[Albe et al., 1990]	$K_{m_{ATP_7}}$	0.12 mM	[Serina et al., 1995]
ASP	4.2 mM	[Bennett et al., 2009]	k_{8f}	16	[Roisin&Kepes, 1978]
$K_{m_{asp_2}}$	21 mM	[Hack et al., 2000]	$K_{m_{UDP_8}}$	0.47 mM	[Roisin&Kepes, 1978]
$h_{asp_{21}}$	2.3	[Xu&Kantrowitz, 1991]	$K_{m_{ATP_8}}$	1.43 mM	[Roisin&Kepes, 1978]
$h_{asp_{22}}$	3.1	[Xu&Kantrowitz, 1991]*	k_{9f}	5.9 sec^{-1}	[MacDonnell et al., 2004]
$K_{m_{CAP_2}}$	0.45 mM	[Shepherdson and Pardee, 1962]	$K_{m_{UTP_9}}$	0.2 mM	[MacDonnell et al., 2004]
h_{CAP}	2.2	[Xu&Kantrowitz, 1991]	h_{UTP_9}	1.8	[MacDonnell et al., 2004]
δ_{CTP_2}	0.22	[Wales et al., 1999]*	K_{CTP_9}	0.11 mM	[Longs&Pardee, 1967]
K_{CTP_2}	0.06 mM	[Wales et al., 1999]*	K_{ATP_9}	7 mM	[MacDonnell et al., 2004]
δ_{UTP_2}	0.9	[Wales et al., 1999]*	$K_{m_{ATP_9}}$	0.6 mM	[MacDonnell et al., 2004]
K_{UTP_2}	1 mM	[Wales et al., 1999]*	h_{ATP_9}	2.1	[MacDonnell et al., 2004]
δ_{ATP_2}	3.4	[Wales et al., 1999]*	k_{0_9}	0.27	[MacDonnell et al., 2004]
K_{ATP_2}	0.34 mM	[Wales et al., 1999]*	δ_{GTP_9}	10.3	[MacDonnell et al., 2004]
ω_2	3	[Wales et al., 1999]*	GTP	4.9 mM	[Bennett et al., 2009]
k_{3f}	195 sec^{-1}	[Washabaugh&Collins, 1984]	$K_{GTP_{91}}$	0.023 mM	[MacDonnell et al., 2004]
$K_{m_{CAASP_3}}$	1.07 mM	[Washabaugh&Collins, 1984]	$\delta_{GTP_{91}}$	0	[MacDonnell et al., 2004]
k_{4f}	222 sec^{-1}	[Björnberg et al., 1999]	$K_{GTP_{92}}$	0.19 mM	[MacDonnell et al., 2004]
Q	0.09 mM	[Shestopalov et al., 1997]	h_{GTP9}	4	[MacDonnell et al., 2004]
K_{m_Q}	0.0394 mM	[Björnberg et al., 1999]	k_{10}	$2*10^{-4}$ sec^{-1}	[Sundararaj et al., 2004, Bremer&Dennis, 1996, Karr et al., 2012]
$K_{m_{DOROA_4}}$	0.0288 mM	[Björnberg et al., 1999]	k_{11}	$2*10^{-4}$ sec^{-1}	[Sundararaj et al., 2004, Bremer&Dennis, 1996, Karr et al., 2012]
k_{4r}	10 sec^{-1}	**	k_{22}	$3*10^{-4}$ sec^{-1}	[Bremer&Dennis, 1996]
			k_{23}	$3*10^{-4}$ sec^{-1}	[Bremer&Dennis, 1996]

** Parameters adapted to the experimental data from the indicated published data.*

*** Parameters that were estimated based on known kinetic parameters for this reaction.*

References cited in the Supplementary Text S1

1. Albe K.R., Butler M.H. and Wright B.E. 1990. Cellular concentrations of enzymes and their substrates. *J. Theor. Biol.* 143, 163-195.
2. Anderson P.M., Meister A. 1966. Control of *Escherichia coli* carbamyl phosphate synthetase by purine and pyrimidine nucleotides. *Biochemistry*. 5, 3164-3169.
3. Bekker M., Kramer G., Hartog A.F., Wagner M.J., de Koster C.G., Hellingwerf K.J. and Teixeira de Mattos M.J. 2007. Changes in the redox state and composition of the quinone pool of *Escherichia coli* during aerobic batch-culture growth. *Microbiology*, 153, 1974–1980.
4. Bennett B.D., Kimball E.H., Gao M., Osterhout R., Van Dien S.J., Rabinowitz J.D. 2009. Absolute metabolite concentrations and implied enzyme active site occupancy in *Escherichia coli*. *Nat. Chem. Biol.* 5, 593-599.
5. Björnberg O., Grüner A.C., Roepstorff P., Jensen K.F. 1999. The activity of *Escherichia coli* dihydroorotate dehydrogenase is dependent on a conserved loop identified by sequence homology, mutagenesis, and limited proteolysis. *Biochemistry*. 38, 2899-2908.
6. Boettcher B., Meister A. Regulation of *Escherichia coli* carbamyl phosphate synthetase. 1982. Evidence for overlap of the allosteric nucleotide binding sites. *J.Biol.Chem.* 257(23), 13971-13976.
7. Bremer H., Dennis P. P. 1996. Modulation of chemical composition and other parameters of the cell by growth rate. Neidhardt et al. eds. *Escherichia coli* and *Salmonella typhimurium*: Cellular and Molecular Biology, 2nd ed. chapter 97.
8. Briozzo P., Evrin C., Meyer P., Assairi L., Joly N., Barzu O., Gilles A.M. 2005. Structure of *Escherichia coli* UMP kinase differs from that of other nucleoside monophosphate kinases and sheds new light on enzyme regulation. *J. Biol. Chem.* 280, 25533-25540.
9. Changeux JP, Gerhart JC, Schachman HK. 1968. Allosteric interactions in aspartate transcarbamylase. I. Binding of specific ligands to the native enzyme and its isolated subunits. *Biochemistry*. 7(2), 531-8. doi: 10.1021/bi00842a007.
10. Donovan W.P., Kushner S.R. 1983. Purification and characterization of orotidine-5'-phosphate decarboxylase from *Escherichia coli* K-12. *J. Bacteriol.* 156, 620-624.
11. Eroglu B, Powers-Lee SG. 2002. Unmasking a functional allosteric domain in an allosterically nonresponsive carbamoyl-phosphate synthetase. *J Biol Chem.* 277(47), 45466-72. doi: 10.1074/jbc.M208185200.
12. Fresquet V, Mora P, Rochera L, Ramón-Maiques S, Rubio V, Cervera J. 2000. Site-directed mutagenesis of the regulatory domain of Escherichia coli carbamoyl phosphate synthetase identifies crucial residues for allosteric regulation and for transduction of the regulatory signals. *J Mol Biol.* 299(4), 979-91. doi: 10.1006/jmbi.2000.3794
13. Hack E.S., Vorobyova T., Sakash J.B., West J.M., Macol C.P., Hervé G., Williams M.K., Kantrowitz E.R. 2000. Characterization of the aspartate transcarbamoylase from *Methanococcus jannaschii*. *J Biol Chem.* 275(21), 15820-7. doi: 10.1074/jbc.M909220199.

14. Holden H.M., Thoden J.B., Raushel F.M. 1999. Carbamoyl phosphate synthetase: an amazing biochemical odyssey from substrate to product. *Cell Mol Life Sci.* 56(5-6), 507-22. doi: 10.1007/s000180050448.
15. Karr J.R., Sanghvi J.C., Macklin D.N., Gutschow M.V., Jacobs J.M., Bolival B.Jr., Assad-Garcia N., Glass J.I. and Covert M.W. 2012. A whole-cell computational model predicts phenotype from genotype. *Cell*, 150, 389–401.
16. Likhoshvai V., Ratushny A. 2007. Generalized Hill function method for modeling molecular processes. *J Bioinform Comput Biol*. 5(2B), 521-31. doi: 10.1142/s0219720007002837.
17. Longs C.W. , Pardee A.B. 1967. Cytidine triphosphate synthetase of *Escherichia coli B* I. Purification and kinetics. *J. Biol. Chem.*, 242(20), 4715-4721.
18. MacDonnell J.E, Lunn F.A., Bearne S.L. 2004. Inhibition of *E. coli* CTP synthase by the "positive" allosteric effector GTP. Biochim. Biophys. Acta. 1699, 213-220.
19. Meyer P., Evrin C., Briozzo P., Joly N., Bârzu O., Gilles A.M. 2008. Structural and functional characterization of *Escherichia coli* UMP kinase in complex with its allosteric regulator GTP. *J Biol Chem.* 283(51), 36011-8. doi: 10.1074/jbc.M802614200.
20. Pierrat O.A., Javid-Majd F. and Raushel F.M. 2002. Dissection of the conduit for allosteric control of carbamoyl phosphate synthetase by ornithine. *Archives of Biochemistry and Biophysics,* 400(1), 26–33.
21. Robin J.P., Penverne B., Hervé G. 1989. Carbamoyl phosphate biosynthesis and partition in pyrimidine and arginine pathways of *Escherichia coli. In situ* properties of carbamoyl-phosphate synthase, ornithine transcarbamylase and aspartate transcarbamylase in permeabilized cells. *Eur. J. Biochem.* 183, 519-528.
22. Roisin M.P., Kepes A. 1978. Nucleosidediphosphate kinase of *Escherichia coli*, a periplasmic enzyme. *Biochim. Biophys. Acta.* 526, 418-428.
23. Serina L., Blondin C., Krin E., Sismeiro O., Danchin A., Sakamoto H., Gilles A.M., Bârzu O. 1995. *Escherichia coli* UMP-kinase, a member of the aspartokinase family, is a hexamer regulated by guanine nucleotides and UTP. *Biochemistry*. 34, 5066-5074.
24. Serina L., Bucurenci N., Gilles A.-M., Surewicz W.K., Fabian H., Mantsch H.H., Takahashi M., Petrescu I., Batelier G. and Barzu O. 1996. Structural properties of UMP-Kinase from *Escherichia coli*: modulation of protein solubility by pH and UTP. *Biochemistry*, 35, 7003-7011.
25. Shepherdson M., Pardee A. B. 1962. Aspartare transcarbamoyl from *Escherichia coli*. *Meth. Enzymol*. 5, 925-931.
26. Shestopalov A.I., Bogachev A.V., Murtazina R.A., Viryasov M.B., Skulachev V.P. 1997. Aeration-dependent changes in composition of the quinone pool in *Escherichia coli. FEBS Letters*. 404, 272-274.
27. Shimosaka M., Fukuda Y., Murata K., Kimura A. 1985. Purification and properties of orotate phosphoribosyltransferases from *Escherichia coli* K-12, and its derivative purine-sensitive mutant. *J. Biochem*. 98, 1689-1697.
28. Sundararaj S., Guo A., Habibi-Nazhad B., Rouani M., Stothard P., Ellison M., and Wishart D.S. 2004. The CyberCell Database (CCDB): a comprehensive, self-updating, relational database to coordinate and facilitate *in silico* modeling of *Escherichia coli*. *Nucleic Acids Res.* 32 (Database issue), D293–D295.

29. Thoden J.B., Huang X., Kim J., Raushel F.M., Holden H.M. 2004. Long-range allosteric transitions in carbamoyl phosphate synthetase. *Protein Sci.* 13(9), 2398-405. doi: 10.1110/ps.04822704.
30. Wales M.E., Madison L.L., Glaser S.S., Wild J.R. 1999. Divergent allosteric patterns verify the regulatory paradigm for aspartate transcarbamylase. *J. Mol. Biol.* 294, 1387-1400.
31. Washabaugh M.W., Collins K.D. 1984. Dihydroorotase from *Escherichia coli*. *J. Biol. Chem.*, 259(5), 3293-3298.
32. Wild J.R., Loughrey-Chen S.J., Corder T.S. In the presence of CTP, UTP becomes an allosteric inhibitor of aspartate transcarbamoylase. *Proc Natl Acad Sci USA.* 1989.86, 46-50.
33. Xu W., Kantrowitz E.R. 1991. Function of serine-52 and serine-80 in the catalytic mechanism of *Escherichia coli* aspartate transcarbamoylase. *Biochemistry*. 30, 2535-2542.

Supplementary Text S2.

Sensitivity analysis of the model in BioUML

Sensitivity analysis [1] implemented in the platform [2] was utilized to investigate the effect of the parameter change on the model solution considering concentrations of all substances in the biosynthesis pathway as target variables (Figure 1: $x_1, x_2, \dots, x_9$). According to the basic, the method calculates sensitivity measures associated with the steady state of a spatially homogeneous reaction system:

$$\frac{dc}{dt} = f(c,k,t) \;\; c(0) = c^0$$

where $c_{ss}(k_i)$ and $c_{ss}(k_i + \triangle k_i)$ indicate solutions of the algebraic systems $f(c,k_i)$ and $f(c,k_i + \triangle k_i)$, correspondingly, while the scaled sensitivities are calculated by multiplying each component $\frac{\partial c^\alpha}{\partial k_i}$ of the vector $\frac{\partial c}{\partial k_i}$ by the normalization factor $\frac{k_i}{c^\alpha{}_{ss}(k_i)}$. As a result of the analysis, we used the scaled sensitivity measures (see Supplementary File S5).

Continuation method with respect to the model parameter and Godunov-Bulgakov method guaranteed asymptotic stability of the stationary solution implemented in STEP+ program

The STEP+ program [3] provides a comprehensive study of a mathematical model in the form of an autonomous system composed of n differential equations with the vector of parameters p:

$$\dot{x} = f(x,p) \qquad (1)$$

Here $f(x,p)$ is a sufficiently smooth vector-function of the vector arguments $x \in R^n$ and $p \in R^m$ in the domain of its definition.

The STEP+ comprises algorithms for numerical analysis of the solution of the autonomous system depending on the model parameters: multistep Gear method with a variable order of accuracy for integration of stiff systems [4]; method of solution continuation with respect to a parameter for constructing the stationary solutions diagram of the autonomous system (1) [3, 5]; Godunov-Bulgakov numerical criterion for determining the guaranteed asymptotic stability of stationary solutions, depending on the model parameter $\alpha \in p$ [6].

The continuation method with respect to a parameter is used to study the dependence of the solution of a system of nonlinear equations (not related to the autonomous system):

$$f(x,\alpha) = 0 \qquad (2)$$

on a scalar parameter $\alpha, \alpha \in p \in R^m$.

The plots of one or several components of the vector-function $x = x(\alpha)$ which is the solution of Eq. (2) will be referred to as the diagram of stationary solutions. The method is based on the implicit function theorem. According to this theorem, the plot of the solution of Eq. (2) in the (n+1)-dimensional space will be a smooth space curve if in a neighborhood of the space curve the rank of matrix A ($A = [f_x, f_\alpha]$) of the derivatives of the right-hand sides is always equal to n, regardless of α. Notice that the smooth space curve may intersect the hyperplane $\alpha = \alpha^*, \alpha^* \in [\alpha_0, \alpha_1]$ several times, meaning that there is a multiplicity of solutions of (2) when $\alpha = \alpha^*$. Thus, the continuation method enables to plot a smooth space curve defined by Eq. (2) that may have turning points and, consequently, have several solutions within some ranges of α.

Eventually, Godunov-Bulgakov method [6] implemented in the tool provides a a numeric criterion of the guaranteed asymptotic stability of the stationary solution. The method, which

does not require calculations of eigenvalues of the Jacobi matrix ($J = [f_x]$) of Eq. (1), is based on estimating the norm of the solution H of the Lyapunov matrix equation

$$J^*H + HJ = -E$$

, where E is a unit matrix, J^* is a matrix adjoint to J. Matrix J is a Hurwitz matrix (i.e. the considered stationary solution is asymptotically stable) if H is a Hermitian positive-definite matrix. In this case, H has an integral presentation in which the matrix exponents of J and J^* are used:

$$H = \int_0^\infty \exp{(tJ^T)}\exp{(tJ)}dt$$

The numeric characteristic $k(J)$ of asymptotic stability when J is a Hurwitz matrix has the form:

$$k(J) = 2\|J\| \sup_{v(0)\neq 0} \frac{\int_0^\infty \|v(t)\|^2 dt}{\|v(0)\|^2}, v(t) = \lceil \exp(tJ) \rceil v(0)$$

At each iteration in the process of calculating $k(J)$, the verification for the inequality is carried out: $\bar{k}(J) < k_0$, where $\bar{k}(J)$ is the approximation of $k(J)$at the iteration, and k_0, is a constant whose value depends only on the precision of number representation by the computer. The asymptotic stability is guaranteed if the limiting value $k(J)$ also satisfy this inequality. If the inequality is not satisfied at some iteration, then the guaranteed conclusion about 'practical' instability of the considered stationary solution is given.

References cited in the Supplementary Text S2

1. Rabitz H., Kramer M., Dacol D. 1983. Sensitivity analysis in chemical kinetics. *Annu. Rev. Phys. Chem.* 34, 419–461. https://doi.org/10.1146/annurev.pc.34.100183.002223.
2. Kolpakov F., Akberdin, Kiselev I., Kolmykov S., Kondrakhin Yu., Kulyashov V., Kutumova E., Pintus S., Ryabova A., Sharipov R., Yevshin I., Zhatchenko S., Kel A. 2022. BioUML—towards a universal research platform. *Nucleic Acids Research*. 50(W1), W124–W131, https://doi.org/10.1093/nar/gkac286.
3. Fadeev S. I., Korolev V. K., Gainova I. A., Medvedev A. E. 2006. The package STEP+ for numerical study of autonomous systems arising when modeling dynamics of genetic-molecular systems. In: *Proceedings of the Fifth International Conference on Bioinformatics of Genome Regulation and Structure*, Novosibirsk, Russia. Eds: Kolchanov N, Hofestadt R. 2, 118-120.
4. Gear C. W. 1971. The automatic integration of ordinary differential equations. *Communications of the ACM*. 14(3), 176-179.
5. Fadeev S. I., Kogai V. V. 2004. Using parameter continuation based on the multiple shooting method for numerical research of nonlinear boundary value problems. *International Journal of Pure and Applied Mathematics*. 14, 467-498.
6. Godunov S. K. 1997. *Ordinary differential equations with constant coefficient* (Vol. 169). American Mathematical Soc.

Supplementary Text S3.

Fitting a model to data

Parameters of the model are to be found as the solution of the inverse problem of mathematical modeling. We utilized the DEEP (Differential Evolution Entirely Parallel) method [**Error! Reference source not found.**, **Error! Reference source not found.**] to fit the experimental data. DEEP is an enhanced modification of the stochastic iterative optimization technique named Differential Evolution [**Error! Reference source not found.**]. DEEP was successfully applied to real biological problems [6-10, 13-14].

Firstly, we fitted our model to Bennett dataset [**Error! Reference source not found.**] that includes stationary concentration values for (casp), (dho), (UDP), (UTP) and (CTP). We used the sum of squared differences between model solution and data for times {7000, 5000, 3000, 1000, 500}. The deviation of the rest of components from the mean concentration over all species was also penalized to make fitting more reliable. This dataset also contains 95% confidence intervals (L,U) for these species that were used to in the penalty function (1).

$$P(\theta) = \exp\left(\Delta^2\right) - 1 \qquad \Delta = \begin{cases} u - L, & \text{if} \quad u < L, \\ u - U, & \text{if} \quad u > U, \\ 0, & \text{otherwise.} \end{cases} \tag{1}$$

where u is the solution. The typical solution for Bennett dataset (Table S2) is presented in Fig. S4.

Secondly, we fitted our model to Ishii dataset [**Error! Reference source not found.**] that contains stationary concentration values for (casp), (UMP), (UDP), (UTP) and (CTP) with dilution rate 0.1 h^{-1}. The typical solution for Ishii dataset is presented in Fig. S5.

Table S2. Experimentally measured steady-state concentrations of pyrimidine biosynthesis metabolites [1].

Model variable	Metabolite	Concentration, mM	Confidence intervals (95%), mM
X_2	carbamayl-aspartate	0.59	0.364 to 0.955
X_3	dihydroorotate	0.0119	0.0116 to 0.0123
X_7	UDP	1.79	1.18 to 2.72
X_8	UTP	8.29	7.76 to 8.86
X_9	CTP	2.73	2.27 to 3.27

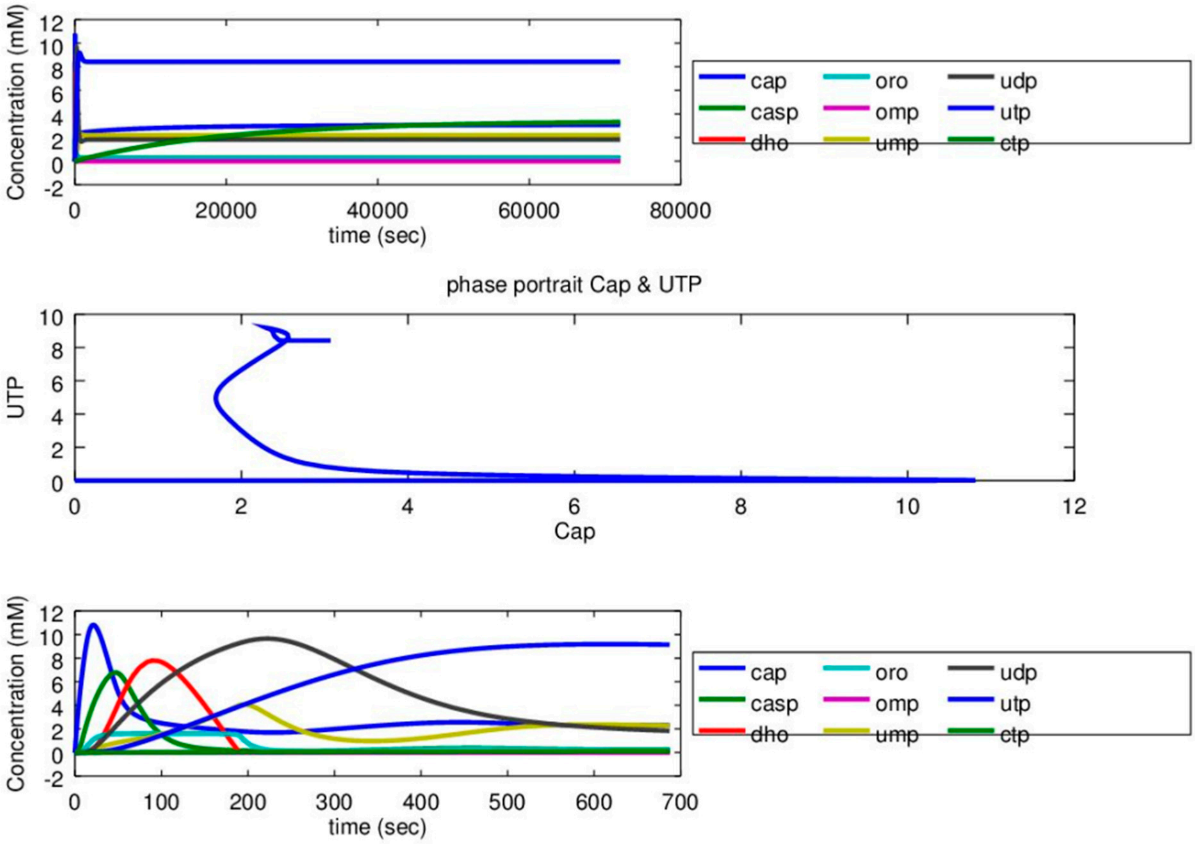

Figure S4. Typical solution for Bennett dataset.

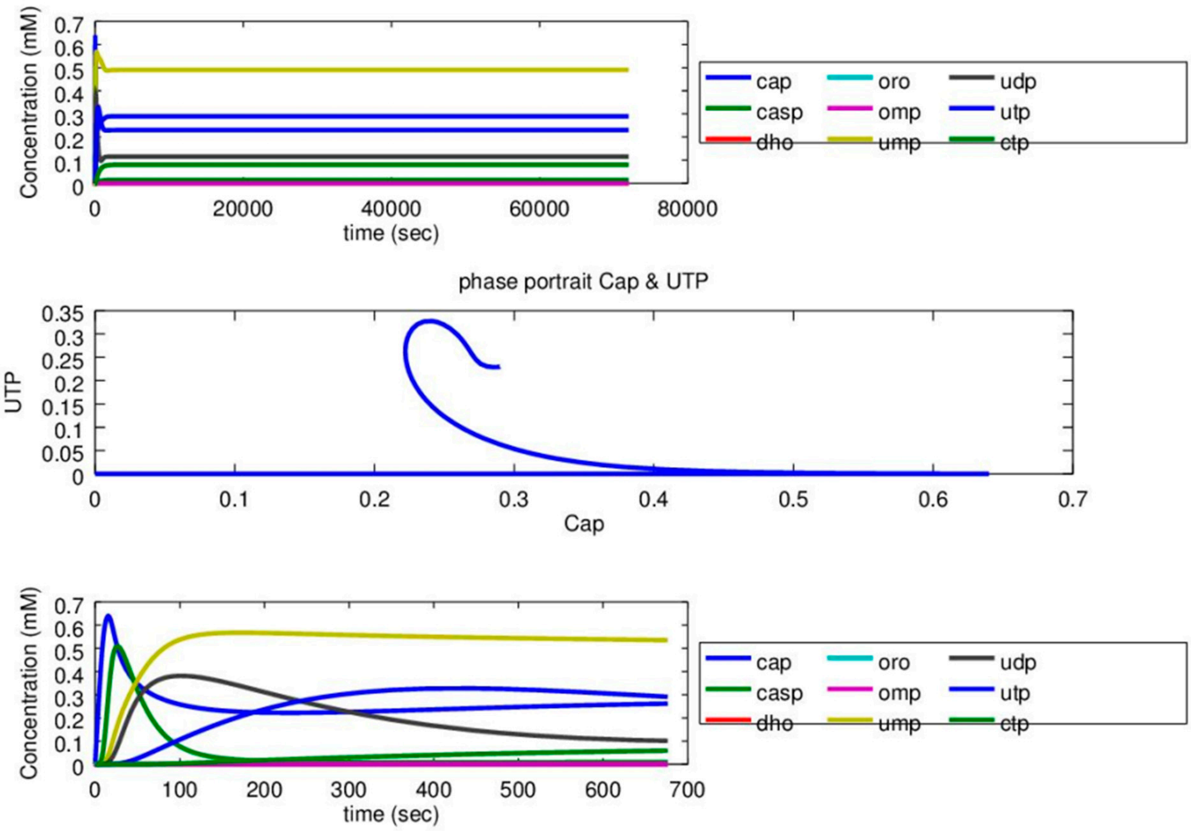

Figure S5. Typical solution for Ishii dataset.

We obtained 10 parameter sets in each run. We compared parameters for two datasets with approximate method of Welch [16], which generalizes the commonly known 2-sample test to the case of arbitrarily many samples. We applied the function *rowFtests* from the *genefilter* package [**Error! Reference source not found.**] from *Bioconductor* [**Error! Reference source not found.**] suite for *R* statistical software.

Таблица S3. Stationary values of concentrations of pyrimidine biosynthesis metabolites measured experimentally [1; 5] and obtained by adapting the complex model by manual fitting and to each of the data sets using the DEEP approach:

Model variableS	Metabolite	Concentration				Manually fitted model	Deep, best solution for Bennet dataset	Deep, best solution for Ishii dataset*
		mM [1]	mM [5]*	mM [5]**	mM [5]***			
X_2	carbamayl-aspartate	0.59	0.242	0.332	0.263	0.0037	0.46	0.19
X_3	Dihydro-orotate	0.0119				0.0004	0.0008	0.0002
X_6	UMP		0.103	0.115	0.228	0.829	0.18	0.038
X_7	UDP	1.79	0.708	0.894	1.513	0.0052	1.57	0.59
X_8	UTP	8.29	0.228	0.165	0.208	5.8858	7.57	3.3
X_9	CTP	2.73	0.104	0.068	0.108	0.0347	2.27	0.74

*— *Steady-state metabolite concentrations (mM) of wild type, cultured at 0.1 h^{-1} dilution rates*
**— *Steady-state metabolite concentrations (mM) of wild type, cultured at 0.4 h^{-1} dilution rates*
***— *Steady-state metabolite concentrations (mM) of wild type, cultured at 0.5 h^{-1} dilution rates*

In Fig. S6 the distributions of four parameters are presented. The difference in *atp* and k_{11} is statistically significant – P=0.02 and P=0.001 respectively. The p-values for *imp* and k_{23} are less significant – 0.2 and 0.05 respectively. We selected these parameters to distinguish strains because they characterize the difference in energy (ATP) and nucleotide pool – purines and pyrimidines – as IMP is the predecessor of purines, k_{11} and k_{23} characterize the outflow of CTP the final product of pyrimidine biosynthesis to RNA and DNA synthesis, correspondingly.

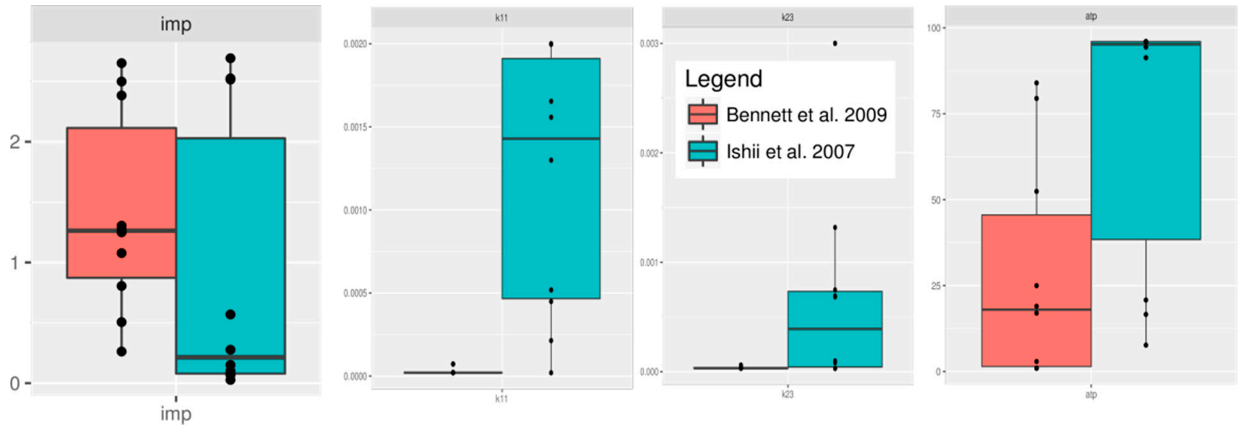

Figure S6. The distributions of four parameters used to distinguish datasets.

Next, we determined the coefficients that characterize the difference in parameters for two datasets. The coefficients are included in the model equations as the multipliers for the corresponding parameters and equal 1 for Bennett dataset and take the values: $Kstrain_{K11}$=70, $Kstrain_{K23}$=12, $Kstrain_{ATP}$=5, $Kstrain_{IMP}$=0.17, for Ishii dataset. The coefficients were obtained as the ratios of the median values for respective runs and rounded to the integer number according to arithmetic rules.

Finally, we fitted the model to both datasets simultaneously (see Fig. S7 for Bennett and Fig. S8 for Ishii). The final mean value ± standard deviation of the objective function was 302.4±9.9. The final value of the objective function for the best parameter set was 296.04 while the value for the manually tuned parameters was as high as 22983.09.

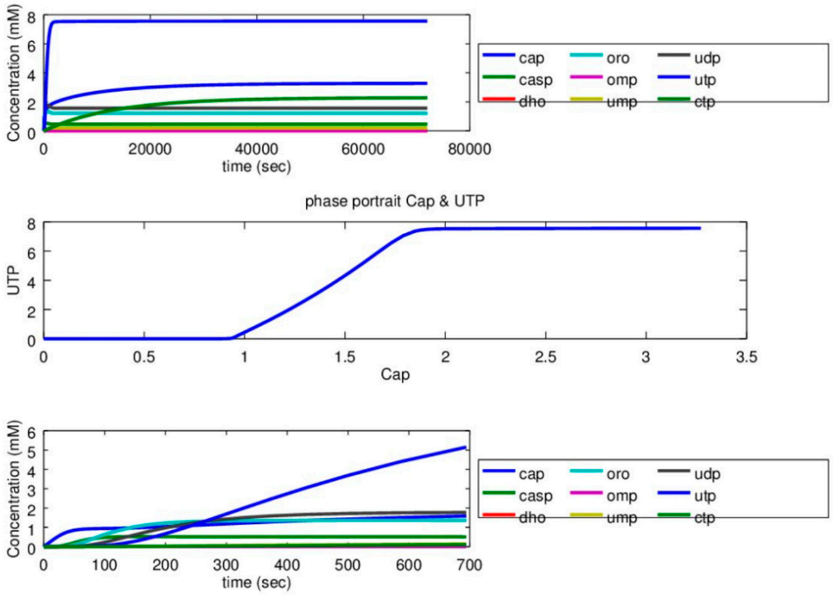

Figure S7. Best solution for Bennett dataset.

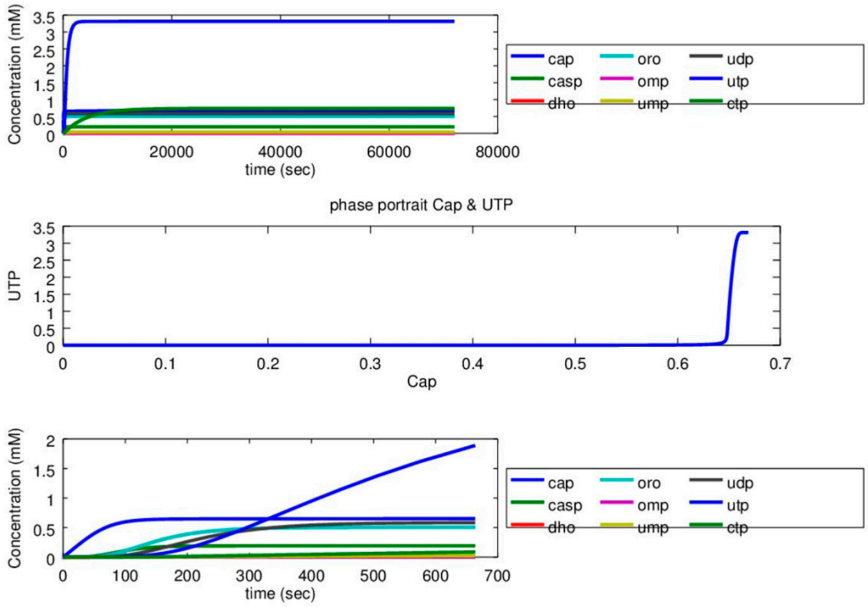

Figure S8. Best model solution for Ishii dataset (0.1 h^{-1} dilution rate).

The confidence intervals and the best values of parameters in respect to the objective function value are presented in Table S4 together with the manually tweaked parameters.

Table S4. The left and right limits of confidence intervals and the values of the best parameters together with the manually tweaked parameters.

Name	left	right	best	manual
k1	0.029249	0.04163	0.038703	0.012
dimp	0.143	0.143	0.143	1.43
kimp1	0.006804	0.565558	0.090293	0.05
kump1	0.152643	0.529068	0.399989	0.04
hump1	3.145288	4.266605	4	1.4
kudp1	6.54435	7.693063	7.299997	0.73
hudp1	3.97213	4.014543	4	1.4
kutp1	10.298915	10.300371	10.299527	1.03
hutp1	3.999839	4.000084	4	1.4
k2	-0.004047	0.172247	0.072903	0.31
kmcap2	1.353958	5.21946	4.5	0.45
hcap	2.77427	4.187238	2.921306	2.2
dctp2	-0.077339	3.388256	2.199982	0.22
kctp2	-0.16269	0.676406	0.096852	0.06
datp2	33.994487	34.002003	33.995005	3.4
katp2	-0.16277	4.803737	2.519826	0.34
dutp2	-0.129867	0.511354	0.09	0.9
kutp2	-2.895075	5.959844	0.1	1
w2	-4.826963	12.847206	0.3	3
k3	-1.258064	3.707315	0.486284	0.351
kmcaasp3	-0.183801	16.379287	10.7	1.07
kf4	1.751338	18.411677	12.699813	1.27
kmdoroa4	-0.023894	0.065757	0.063183	0.0288
kr4	-0.526447	1.045545	0.000111	0.225
kmoroa4	0.895438	1.012971	0.928194	0.01
koroa4	-0.005036	0.185087	0.130715	0.0134
k4	3.130053	3.567362	3.280004	12.28
k5	0.044136	1.052815	0.39	3.9
kmoroa5	0.267614	0.316899	0.3	0.03
k5prpp	68.942271	78.160989	75	7.5
k6	0.06174	0.102693	0.100293	0.73
kmomp6	-0.003444	0.085911	6e-04	0.006
k7	-1.185754	5.271853	0.557148	0.393
kmump7	-0.252322	0.522566	0.5	0.05
r	-0.544994	1.04556	1e-7	1
kutp72	0.560122	1.71142	0.880579	0.15
hutp7	0.854279	1.292012	1.013333	2.4
kutp71	-2.673787	5.629161	0.064403	0.54
k8	0.059097	0.071348	0.061171	0.11

kmudp8	4.68473	4.70623	4.7	0.47
k9	0.000174	0.000174	0.000174	0.0000174
hutp9	3.092223	4.473663	4	1.8
kmutp9	-0.445406	0.911905	0.02	0.2
kctp9	-0.424144	1.549925	1.1	0.11
k10	0.002	0.002	0.002	0.0002
k11	2.7e-05	5.4e-05	4.6e-05	0.0002
k23	3e-05	3.1e-05	3e-05	0.0003
k22	-0.000105	0.00462	0.003	0.0003
atp	-15.743654	57.406474	42.362378	9.6
imp	-0.008816	0.476308	0.074514	0.27

It is evident that manually tweaked and best parameters obtained by fitting are different. Some values, such as *dimp*, $kump_1$, k_{22}, etc, differ by orders of magnitude. That can be explained by the fact that manual tweaking was performed with only Bennett dataset in mind.

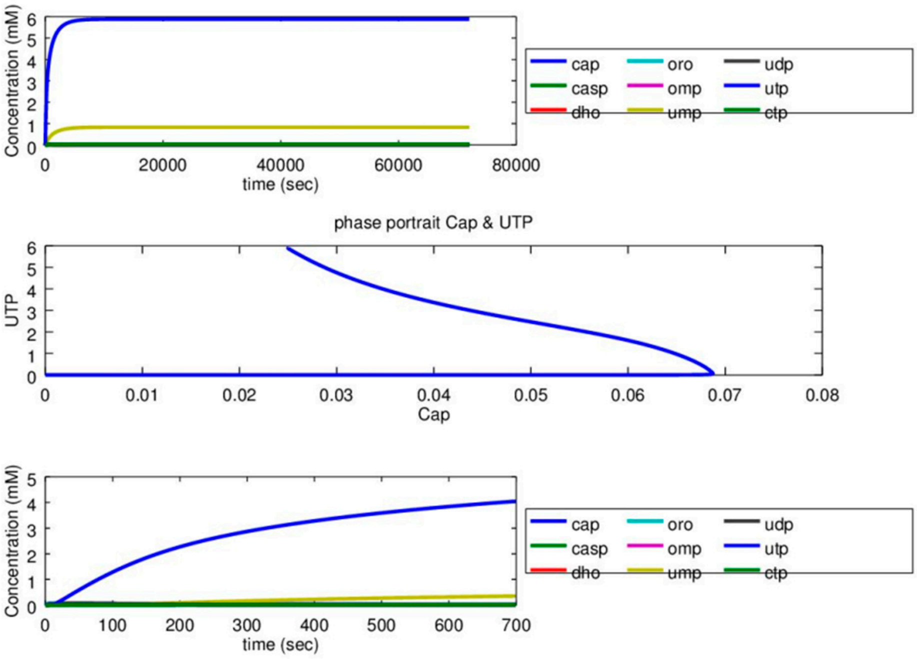

Figure S9. Solution for Bennett with manual parameters.

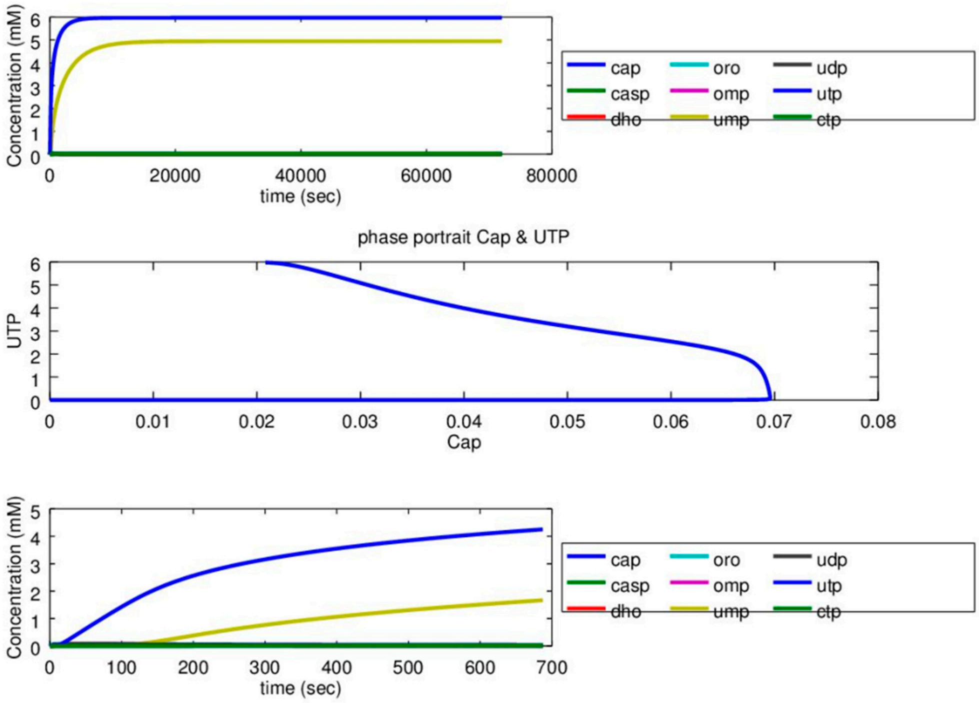

Figure S10. Solution for Ishii with manual parameters.

Supplementary Text S4.

Detection of oscillations in the solution

To detect oscillations in the solution with different parameter combinations automatically we used BaSAR (Bayesian Spectrum Analysis in R), a package for extracting frequency information from time series data [**Error! Reference source not found.**]. The software uses advanced techniques of Bayesian inference that are well suited for handling typical biological data. The core functions are designed for detecting a single key frequency, without the need for data pre-processing such as detrending. The package is freely available at CRAN – The Comprehensive R Archive Network: http://cran.r-project.org/web/packages/BaSAR.

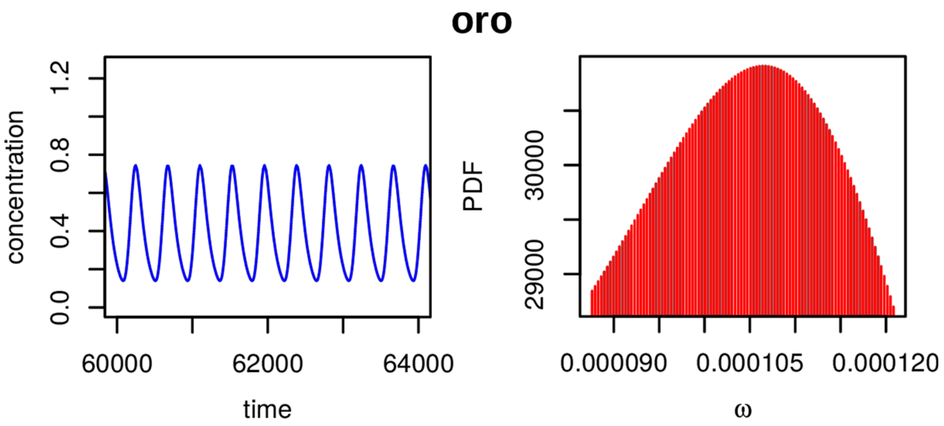

Figure S11. Results of BaSAR application to the model solution (dynamics of orotate concentration). The posterior probability density function (PDF) over the sampled frequency range has a high probability peak and thus confirms oscillatory regime for this parameter set.

We summarise the main points of the methods and refer to [**Error! Reference source not found.**] for further details. We assume that the data are given at discrete time points. There is no requirement for these data to be equally spaced. The data can be modelled as a sum of underlying signal, a background trend, and noise present in the system. The signal can be approximated by a linear combination of model functions, parameterized by the angular frequency. The harmonic functions *sin* and *cos* are used as default model functions. Similarly, any background functions that are present can be approximated by a set of trend functions and Legendre polynomials are used for this purpose. Periodic data and a good model will result in a high probability peak in the posterior distribution at the appropriate frequency.

Application of this approach to the solution with some parameter set results in the graph similar to that shown in Fig. S11.

References cited in the Supplementary Texts S3 and S4

1. Bennett B.D., Kimball E.H., Gao M., Osterhout R., Van Dien S.J., Rabinowitz J.D. 2009. Absolute metabolite concentrations and implied enzyme active site occupancy in *Escherichia coli. Nature Chem. Biol.* 5(8), 593-599.
2. Gentleman R., Carey V., Huber W., Hahne F. 2015. Genefilter: methods for filtering genes from high-throughput experiments. *R package version*, 1(1).
3. Gentleman R.C., Carey V.J., Bates D.M., Bolstad B., Dettling M., Dudoit S., ... & Hornik K. 2004. Bioconductor: open software development for computational biology and bioinformatics. *Genome Biol.* 5(10), R80.
4. Granqvist E., Hartley M., Morris R.J. 2012. BaSAR—A tool in R for frequency detection. *Biosystems*. 110(1), 60-63.
5. Ishii N., Nakahigashi K., Baba T., Robert M., Soga T., Kanai A., ... & Ho P.Y. 2007. Multiple high-throughput analyses monitor the response of *E. coli* to perturbations. *Science*. 316(5824), 593-597.
6. Ivanisenko N.V., Mishchenko E.L., Akberdin I.R., Demenkov P.S., Likhoshvai V.A., Kozlov K.N., ... & Clausznitzer D. 2014. A new stochastic model for subgenomic hepatitis C virus replication considers drug resistant mutants. *PloS one*. 9(3), e91502.
7. Ivanisenko N.V., Mishchenko E.L., Akberdin I.R., Demenkov P.S., Likhoshvai V.A., Kozlov K.N., ... & Ivanisenko V.A. 2013. Replication of the subgenomic hepatitis C virus replicon in the presence of the NS3 protease inhibitors: a stochastic model. *Biophysics.* 58(5), 592.
8. Kozlov K., Gursky V., Kulakovskiy I., Samsonova M. 2014.Sequence-based model of *gap* gene regulatory network. In *Proceedings of BGRS-SB'2014*, Novisibirsk.
9. Kozlov K., Gursky V., Kulakovskiy I., Samsonova M. 2014. Sequence-based model of *gap* gene regulatory network. *BMC genomics*. 15(12), S6.
10. Kozlov K., Gursky V.V., Kulakovskiy I.V., Dymova A., Samsonova M. (2015). Analysis of functional importance of binding sites in the *Drosophila gap* gene network model. *BMC genomics.* 16(13), S7.
11. Kozlov K., Samsonov A. 2011. DEEP—differential evolution entirely parallel method for gene regulatory networks. *The Journal of Supercomp.* 57(2), 172-178.
12. Kozlov K., Samsonov A.M., Samsonova M. 2016. A software for parameter optimization with Differential Evolution Entirely Parallel method. *PeerJ Comp Sci*. 2, e74.
13. Kozlov K., Surkova S., Myasnikova E., Reinitz J., Samsonova M. 2012. Modeling of *gap* gene expression in *Drosophila* Kruppel mutants. *PLoS Comput Biol*. 8(8), e1002635.
14. Nuriddinov M.A., Kazantsev F.V., Rozanov A.S., Kozlov K.N., Peltek S.E., Akberdin I.R., Kolchanov N.A. 2013. Mathematical modeling of ethanol and lactic acid biosynthesis by theromphilic *Geobacillus* bacteria. *Russian Journal of Genetics: Applied Research*. 17(4/1), 686-704.
15. Storn R., Price K. 1997. Differential evolution–a simple and efficient heuristic for global optimization over continuous spaces. *J. Global Optim.* 11(4), 341-359.
16. Welch B.L. 1951. On the Comparison of Several Mean Values: An Alternative Approach. *Biometrika.* 38(3-4), 330-336. http://dx.doi.org/10.1093/biomet/38.3-4.330.
