# Supplementary figures and images for: Impact of Negative Feedbacks on De Novo Pyrimidines Biosynthesis in Escherichia coli
Source: Int J Mol Sci. 2023 Mar 2;24(5):4806. doi: 10.3390/ijms24054806 (PMC10003070; doi:10.3390/ijms24054806)

Model parameters: Bennet (mog) vs Ishi0.1 (mug)

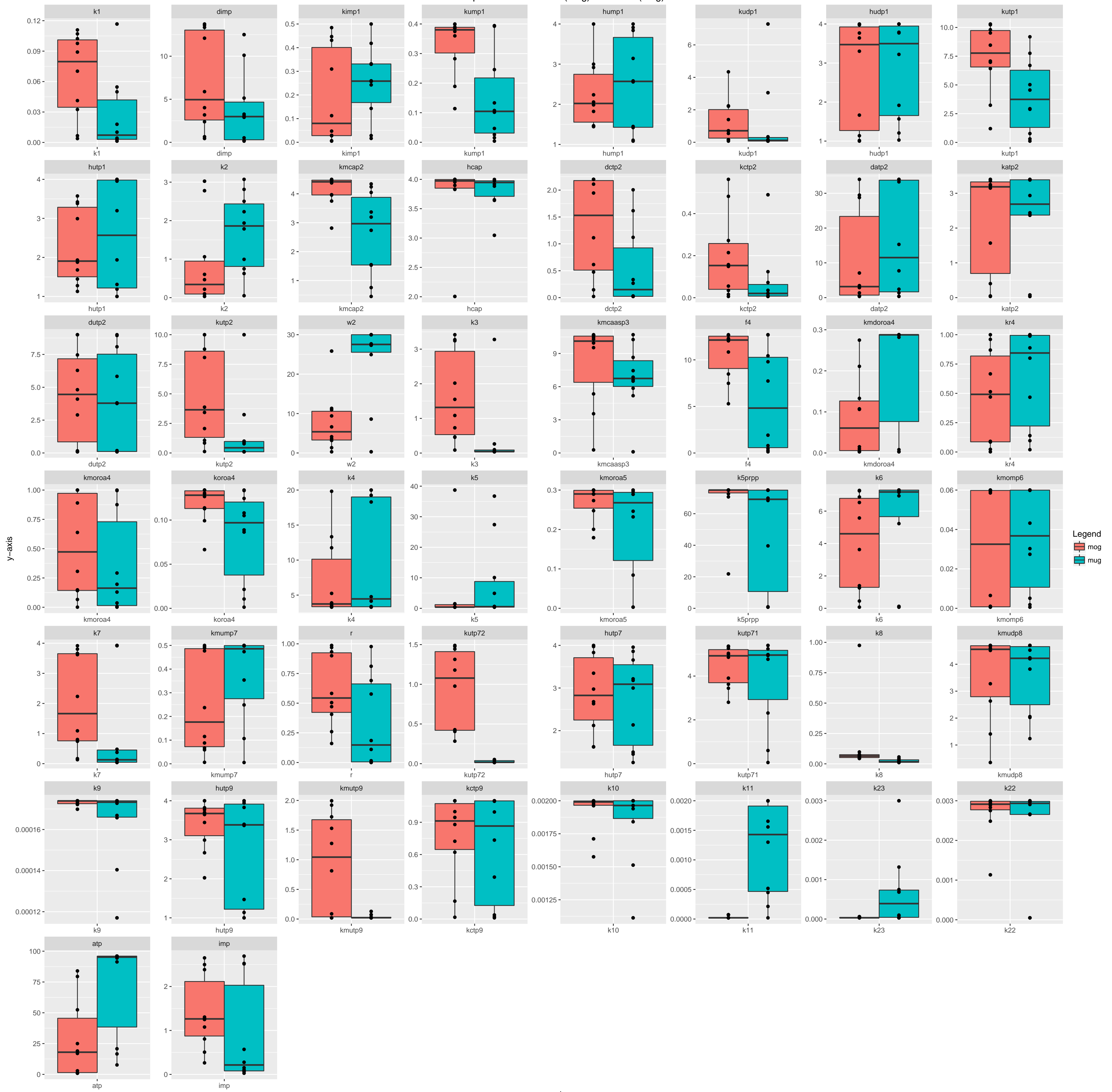

Model parameters: Bennet (mog) vs Ishi0.1 (mug) (P < 0.05)

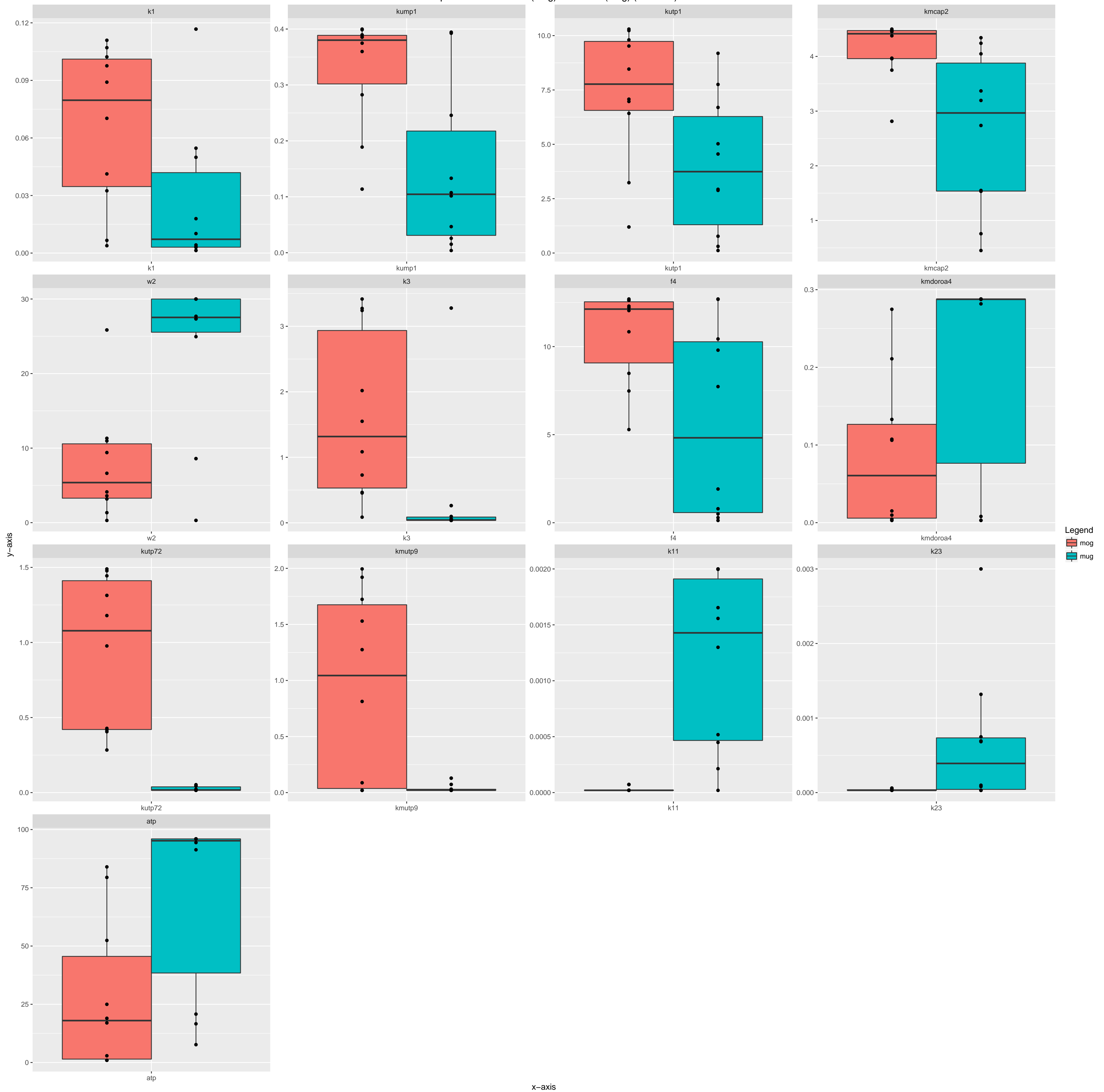

Supplement: Supplementary file 1 [file ijms-24-04806-s001.zip › SF1_Bennet_Ishi_0.1_difference_parameters.pdf]

**p35 = 0**

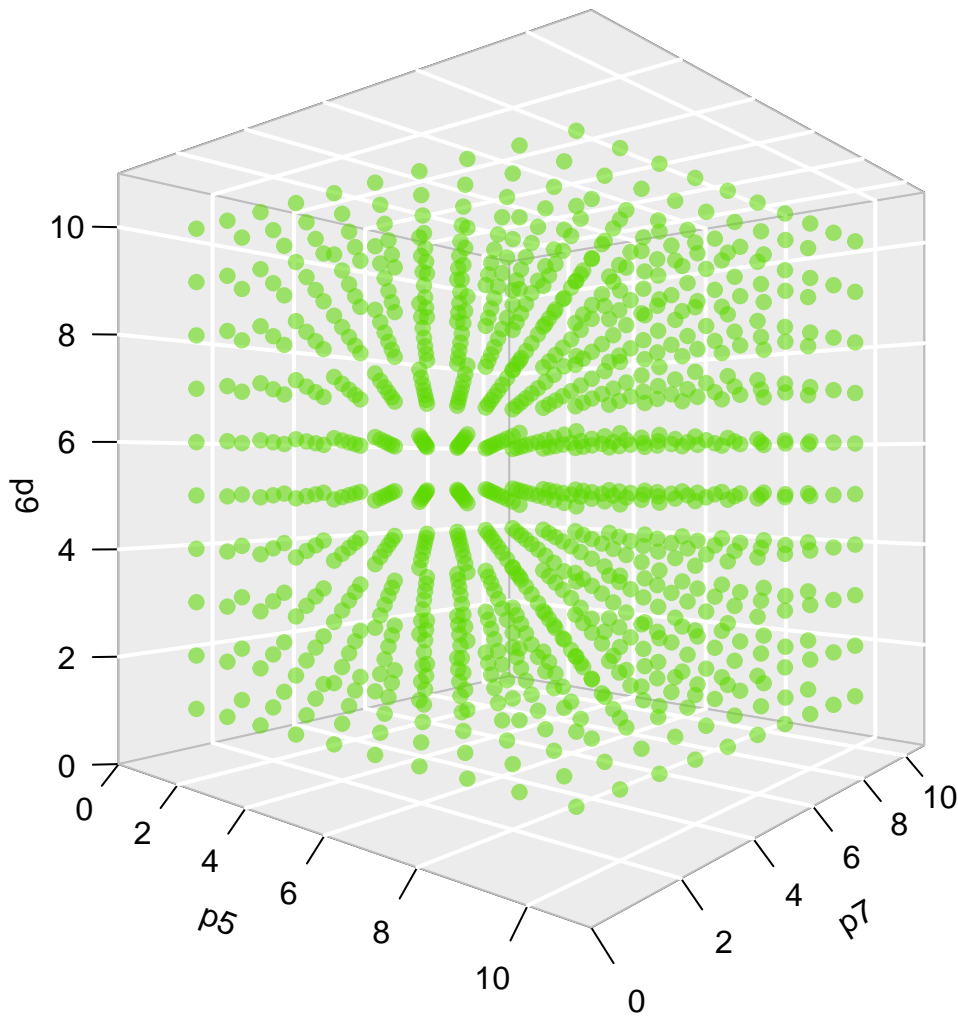

**p35 = 0.07**

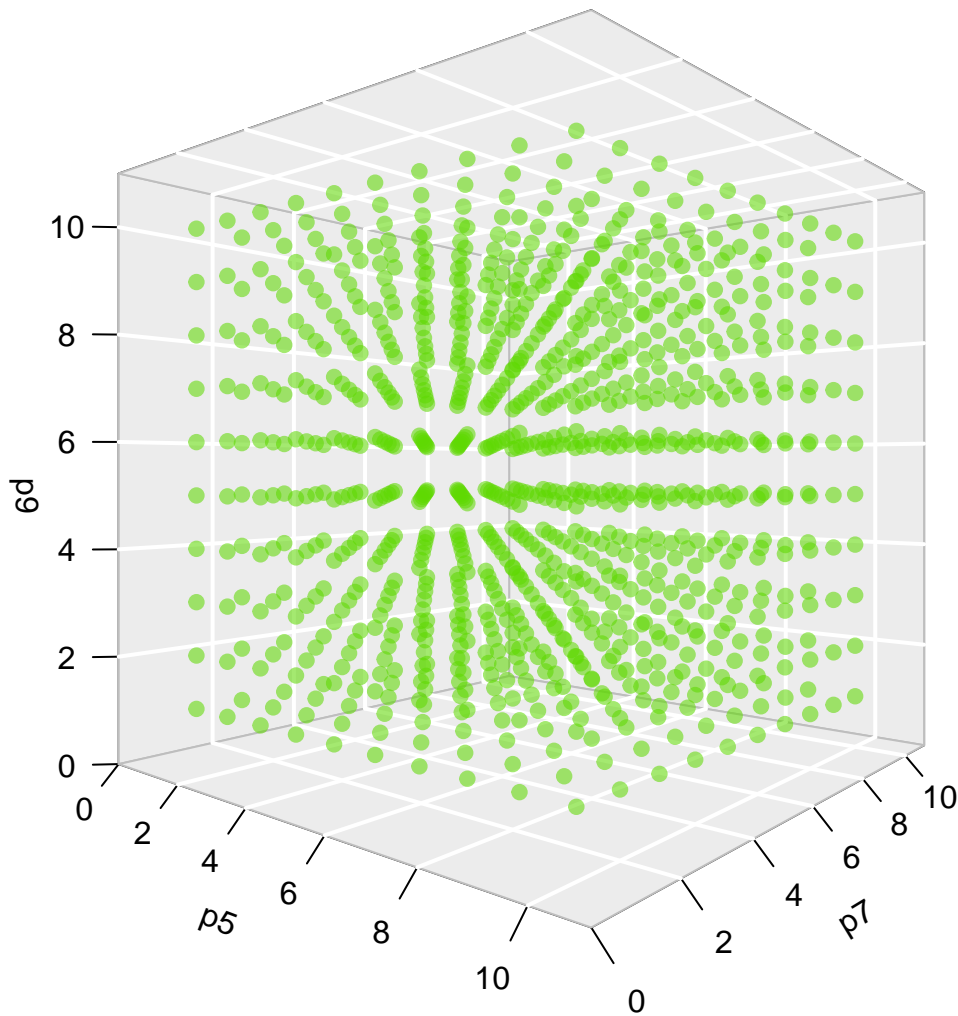

**p35 = 0.14**

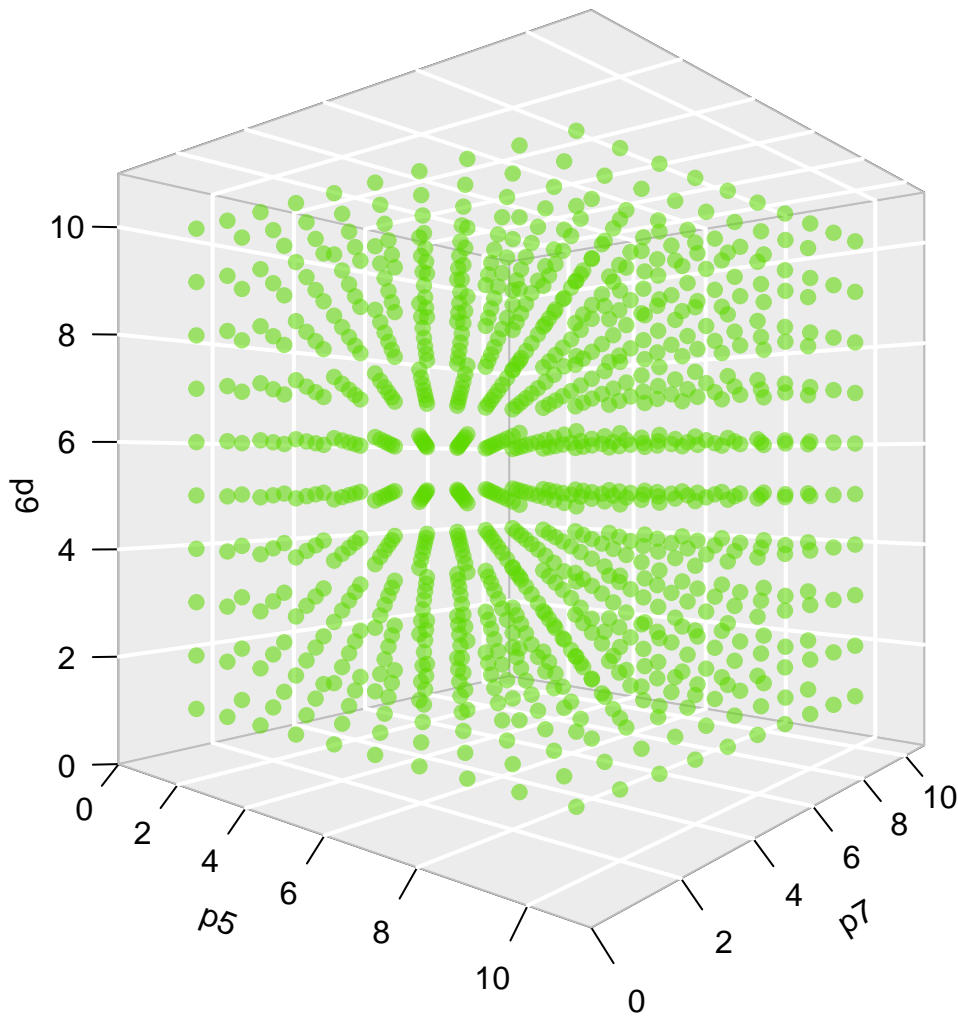

**p35 = 0.21**

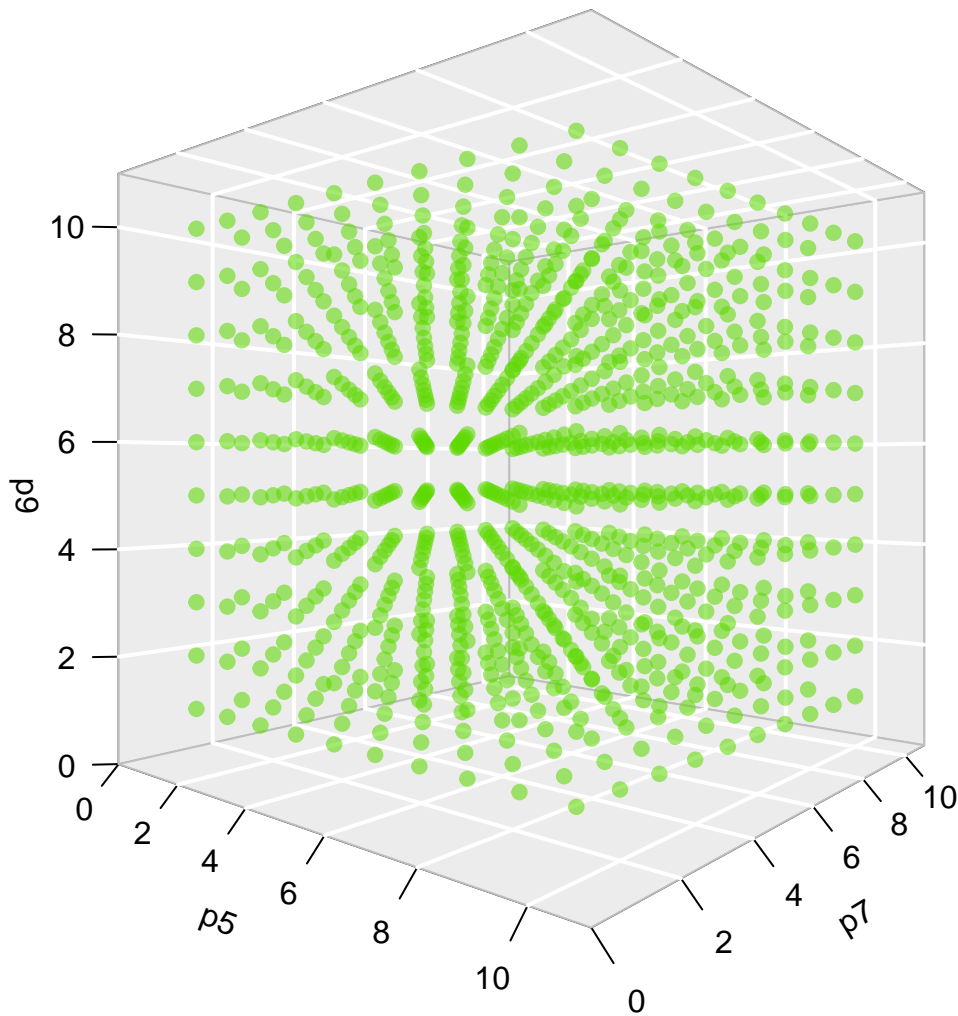

**p35 = 0.28**

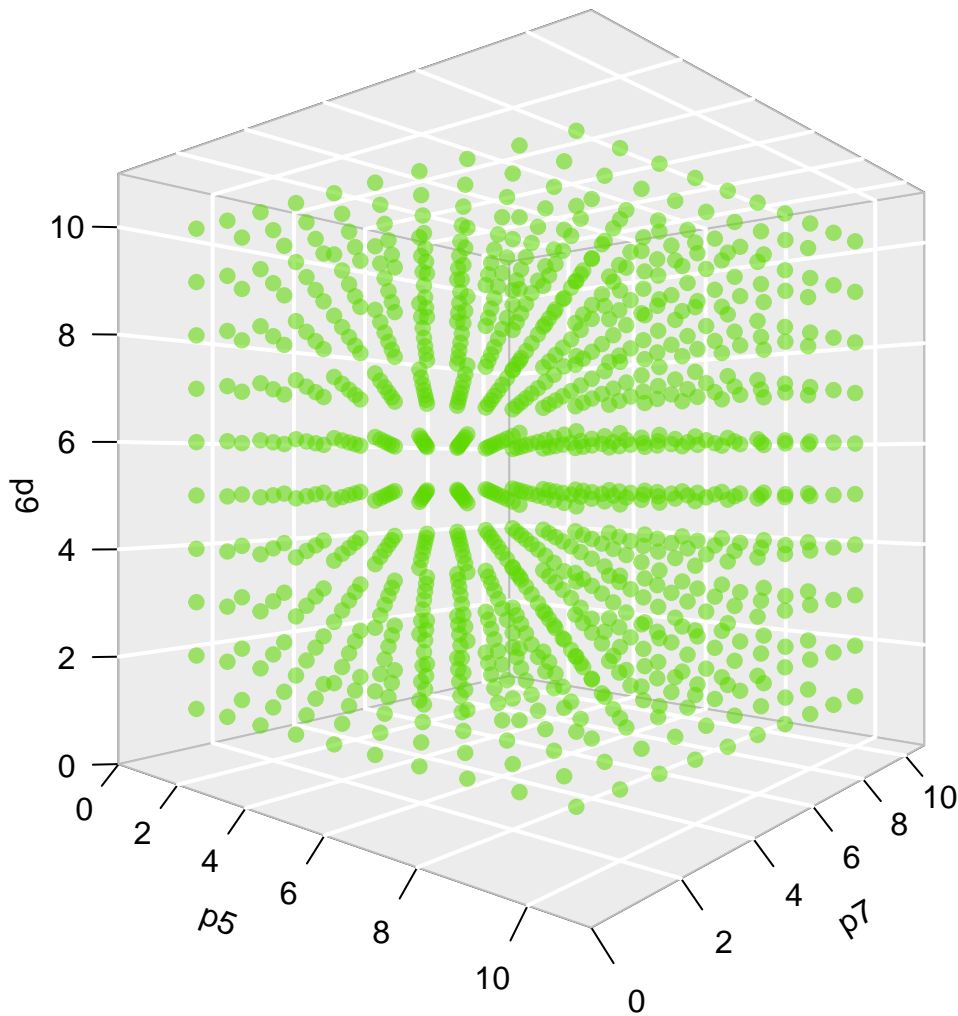

**p35 = 0.35**

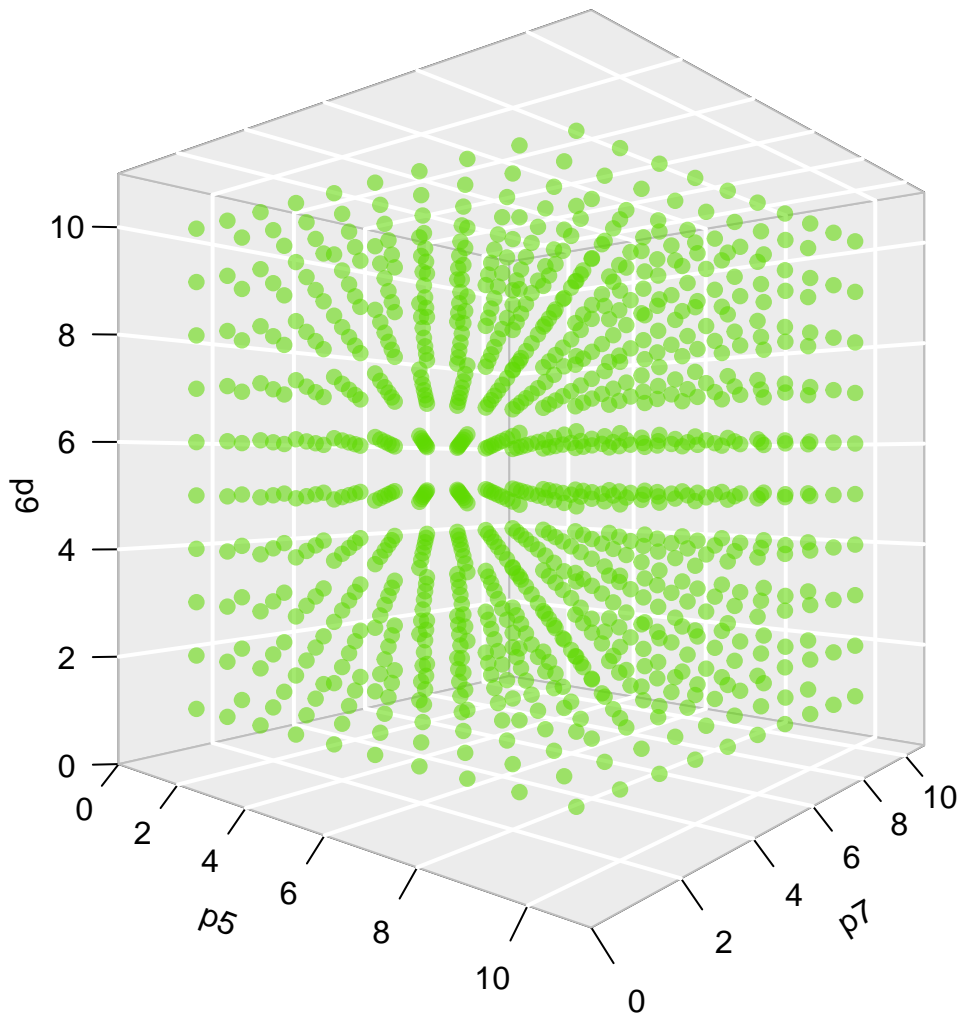

**p35 = 0.42**

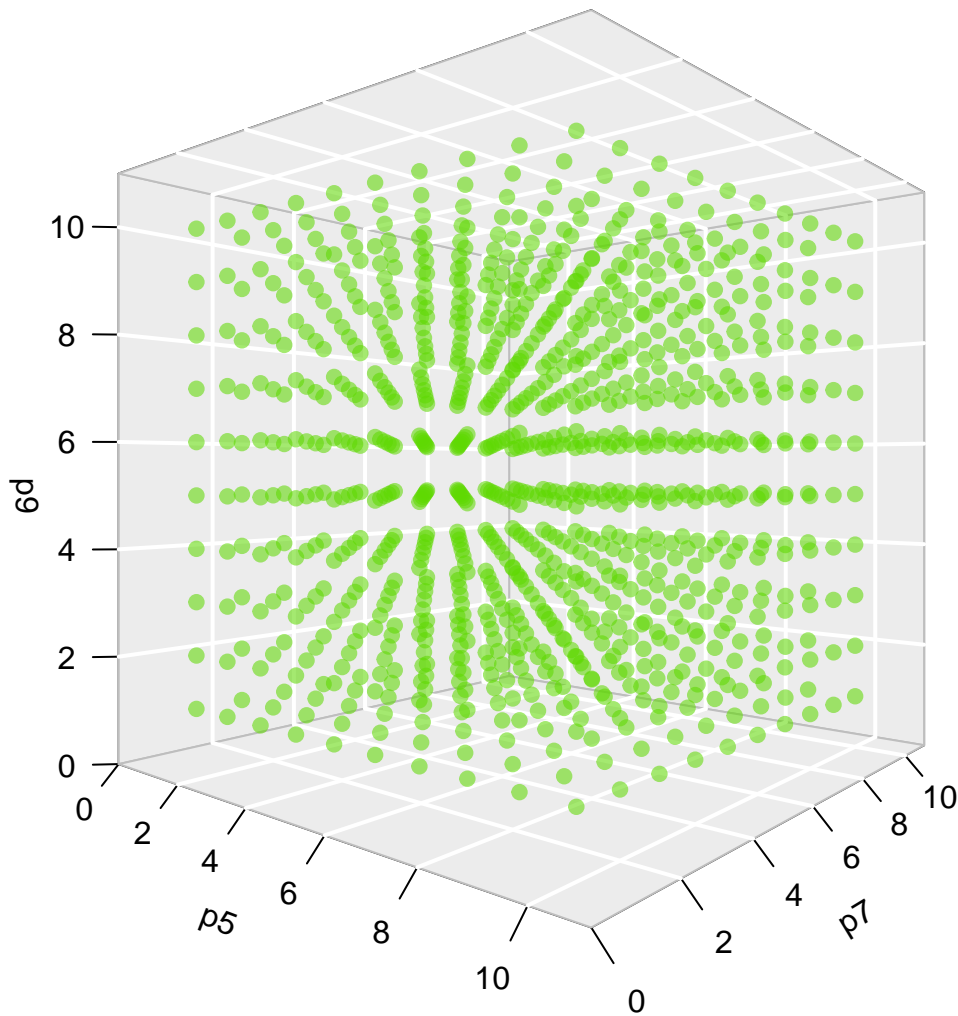

**p35 = 0.56**

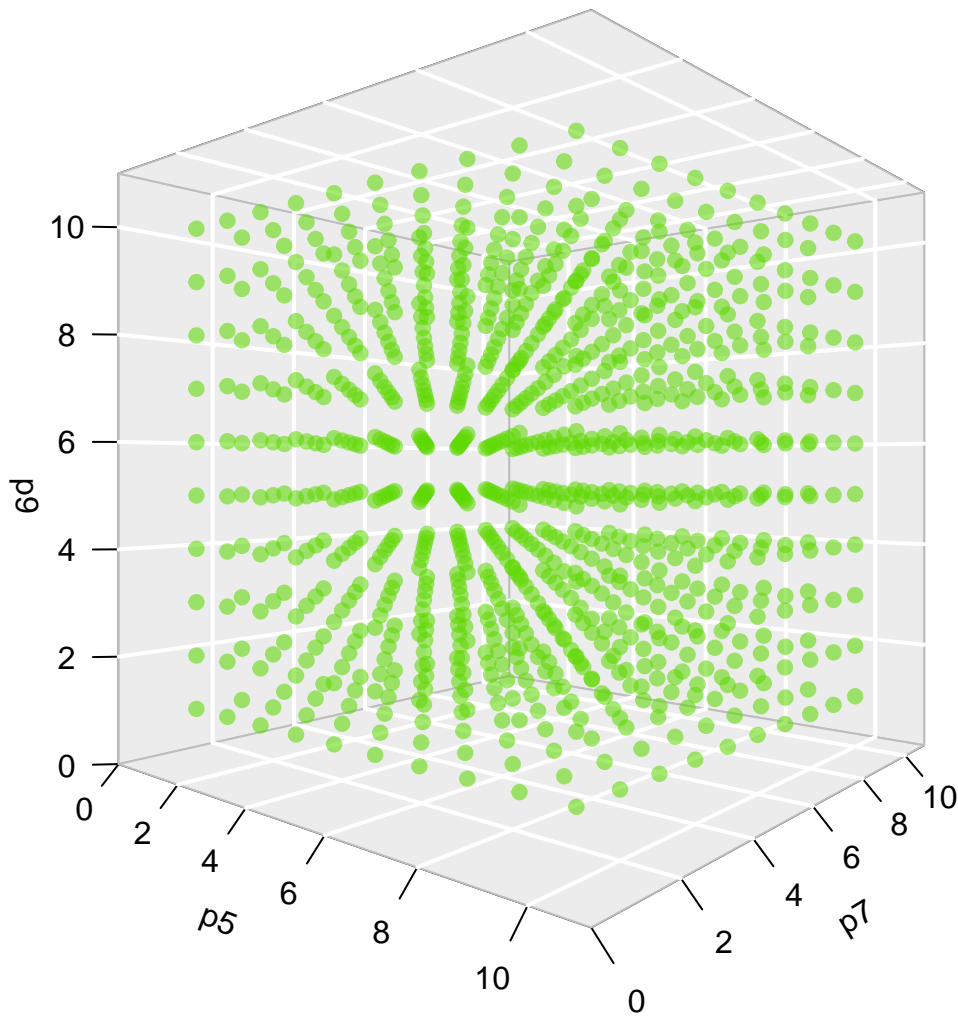

**p35 = 0.63**

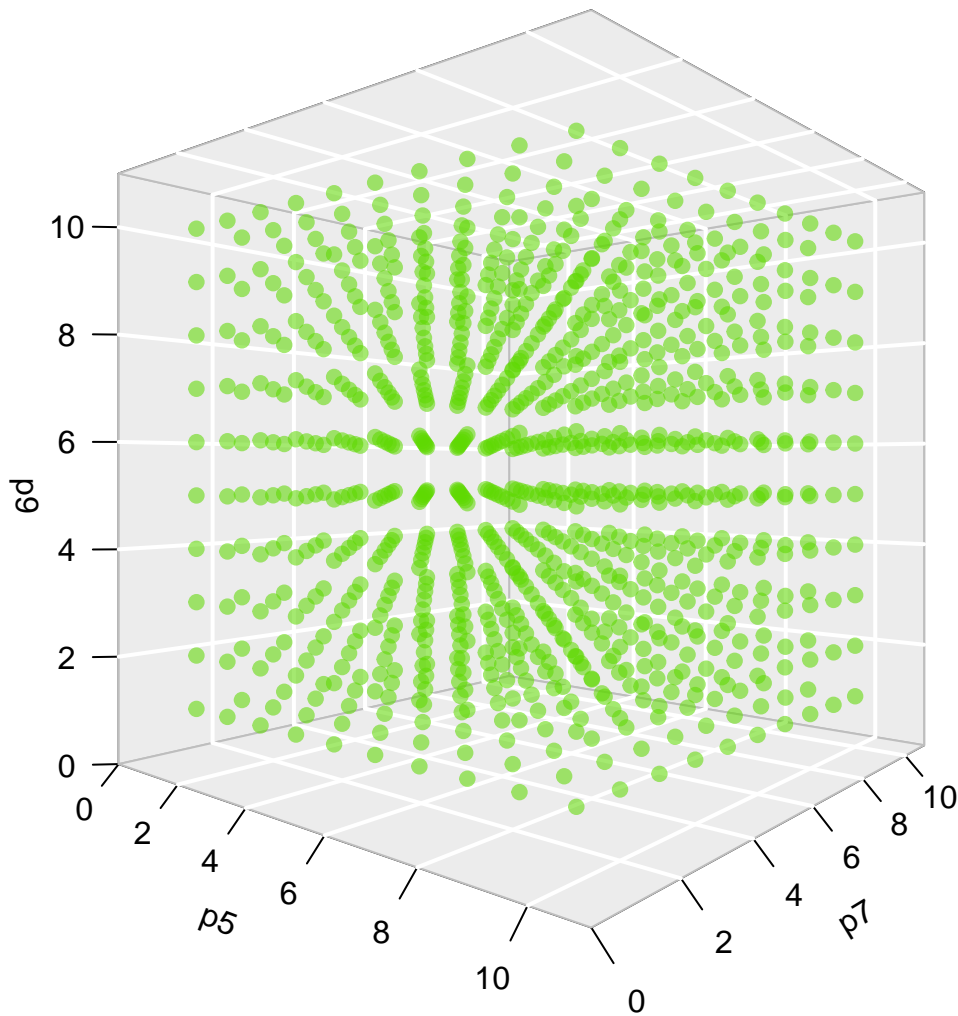

**p35 = 0.85**

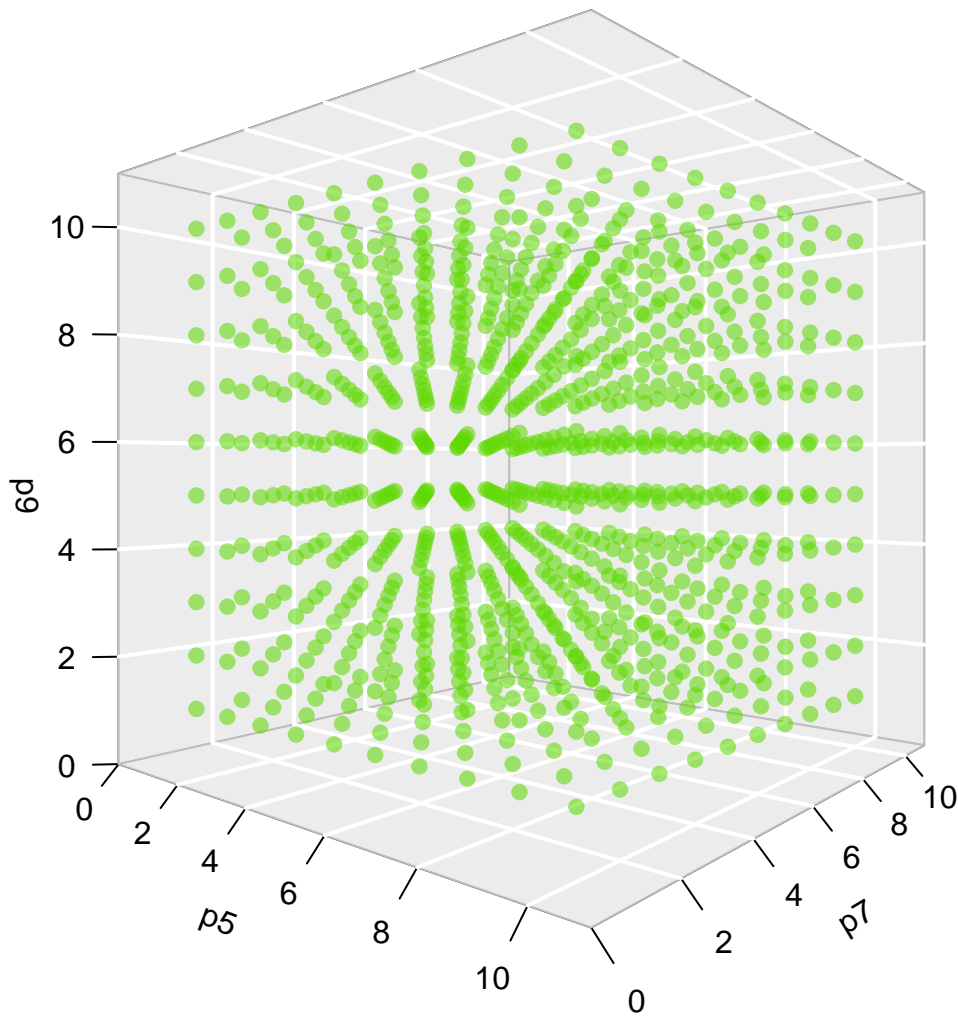

**p35 = 1**

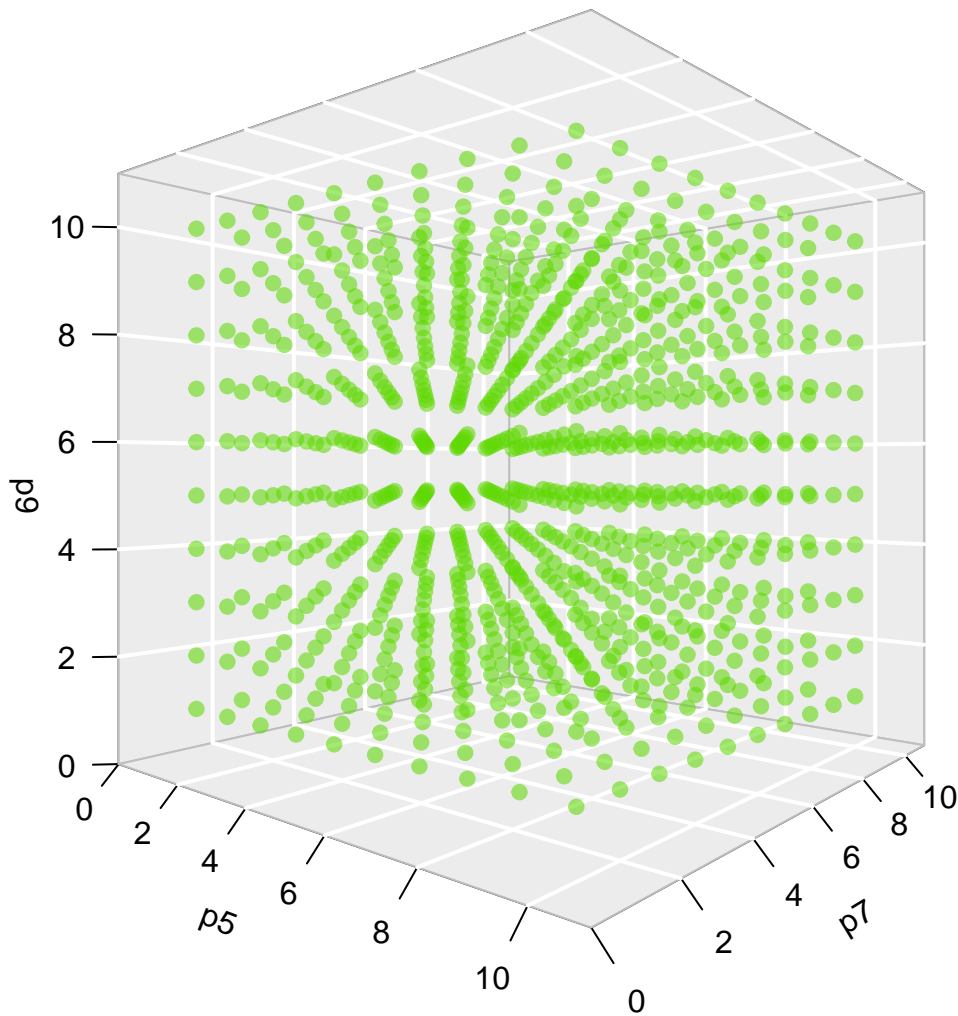

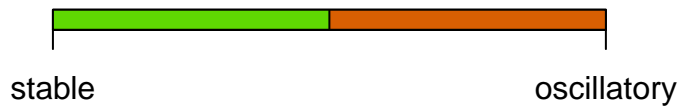

Supplement: Supplementary file 1 [file ijms-24-04806-s001.zip › SF2_Original_model_manual fitted_3Hills_p35_3d.pdf]

**p35 = 0**

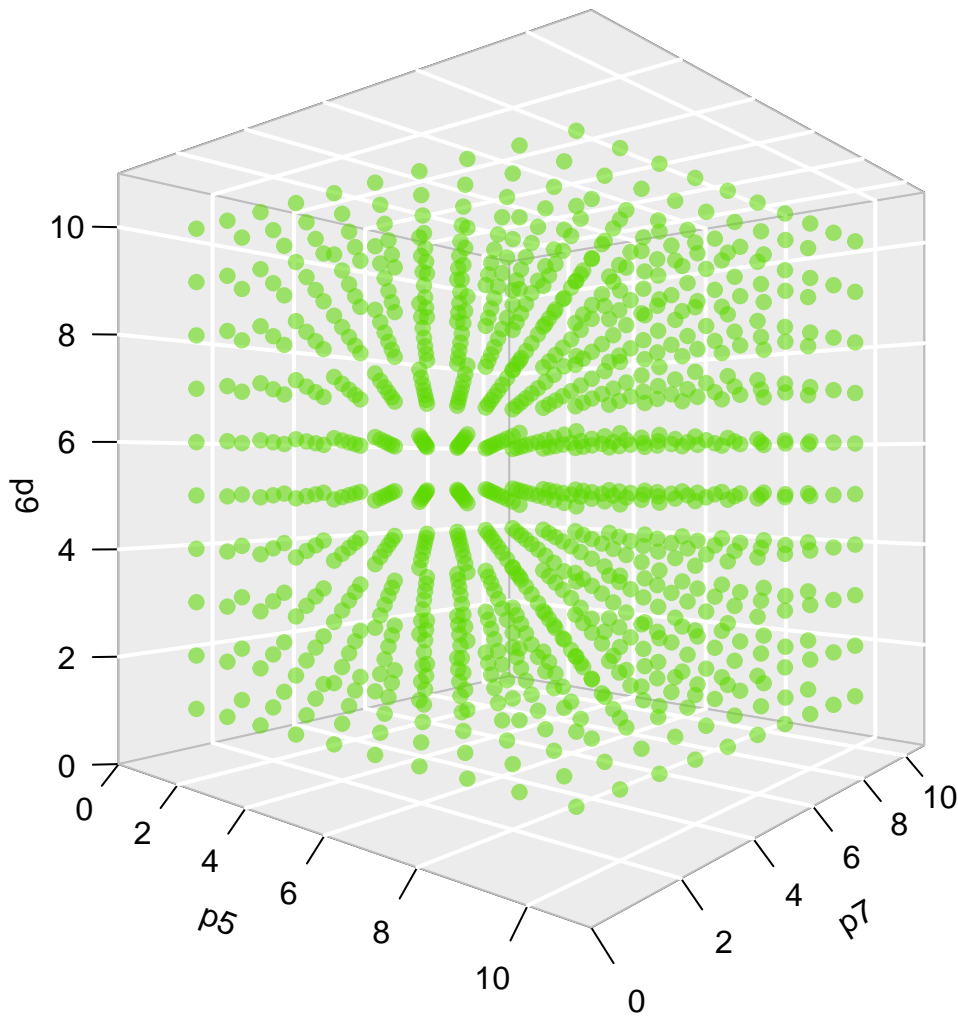

**p35 = 0.07**

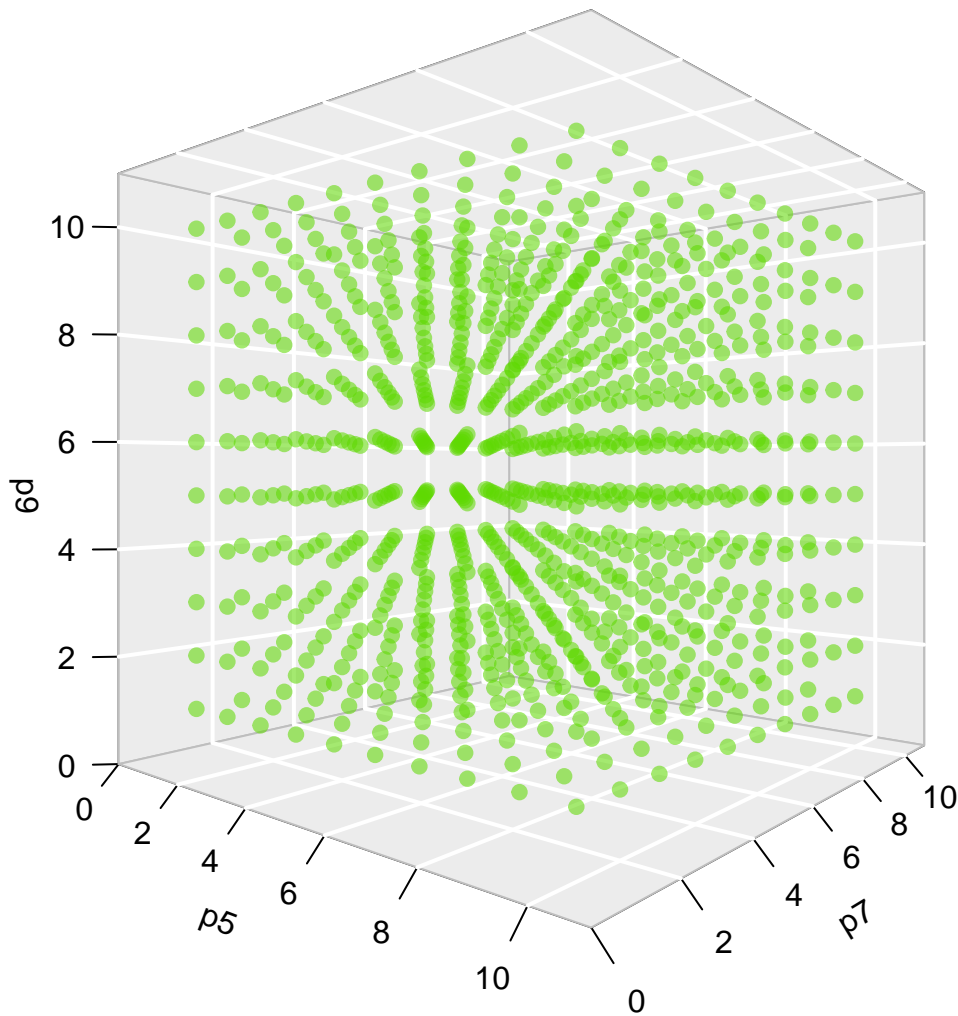

**p35 = 0.14**

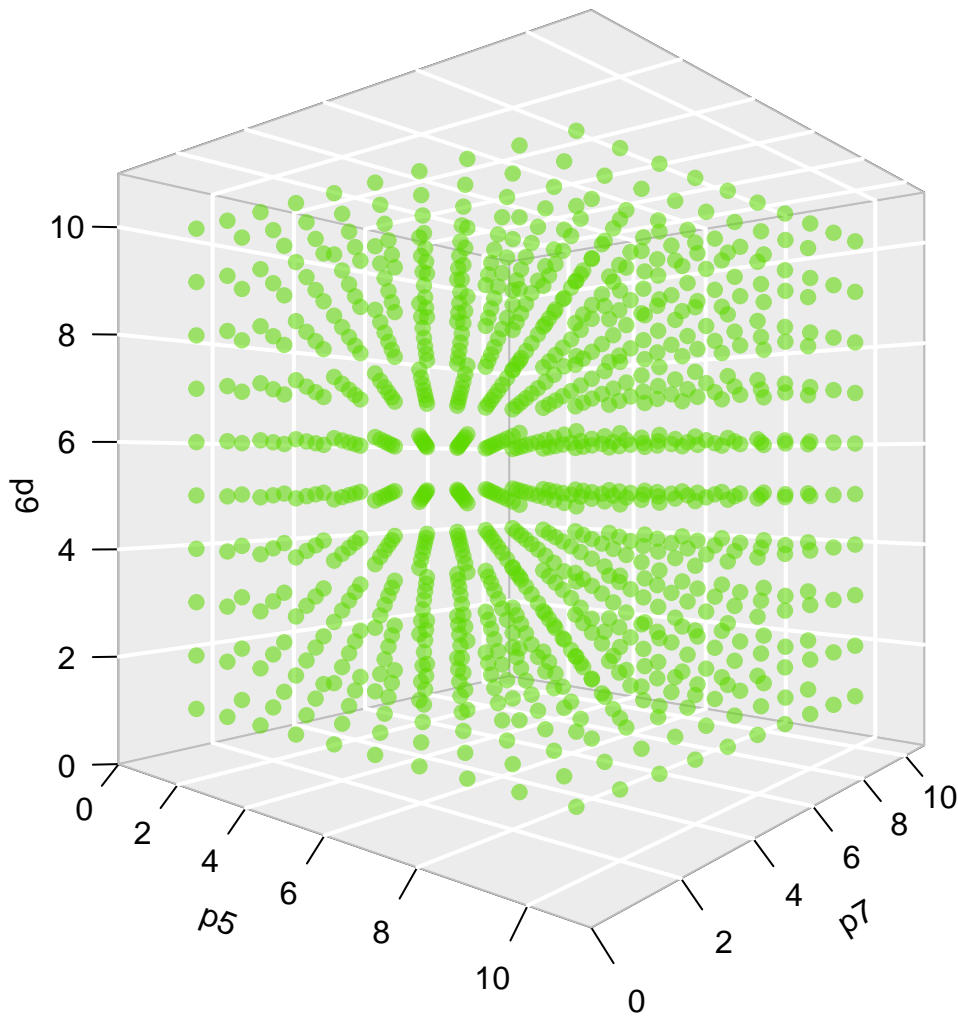

**p35 = 0.21**

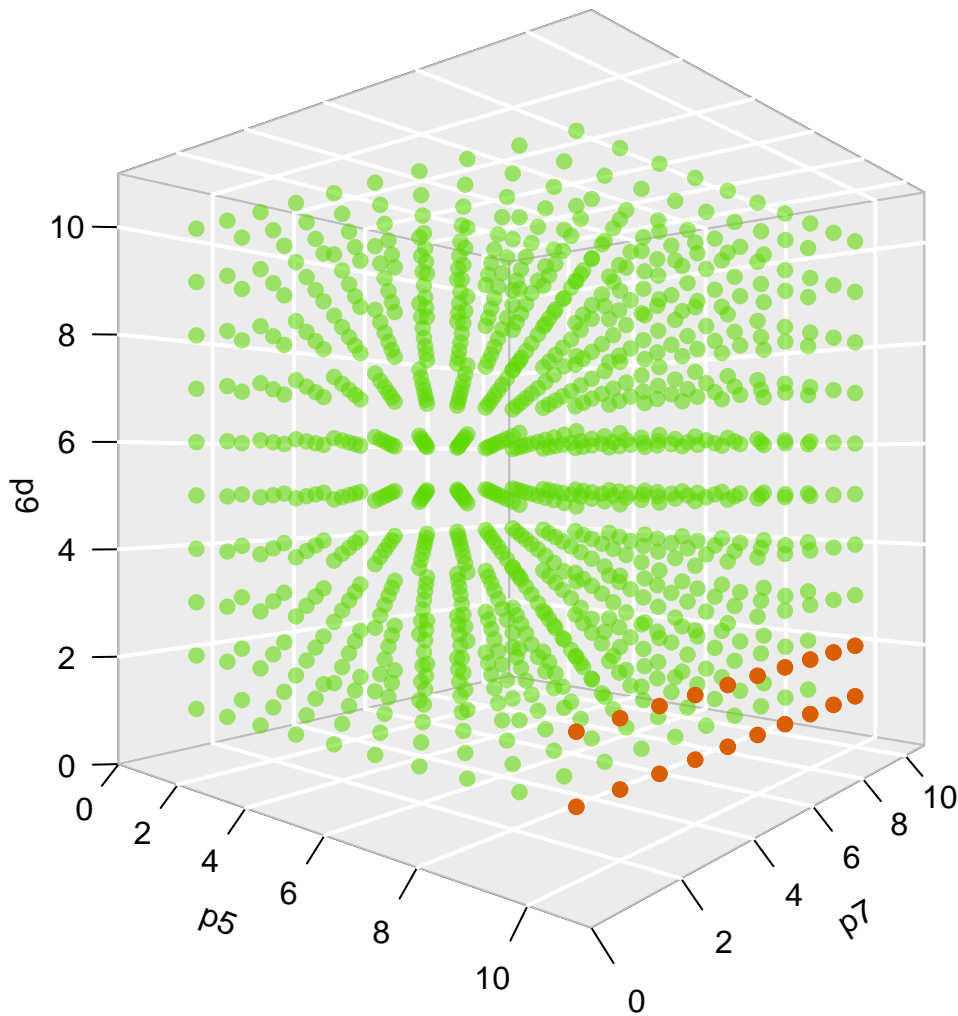

**p35 = 0.28**

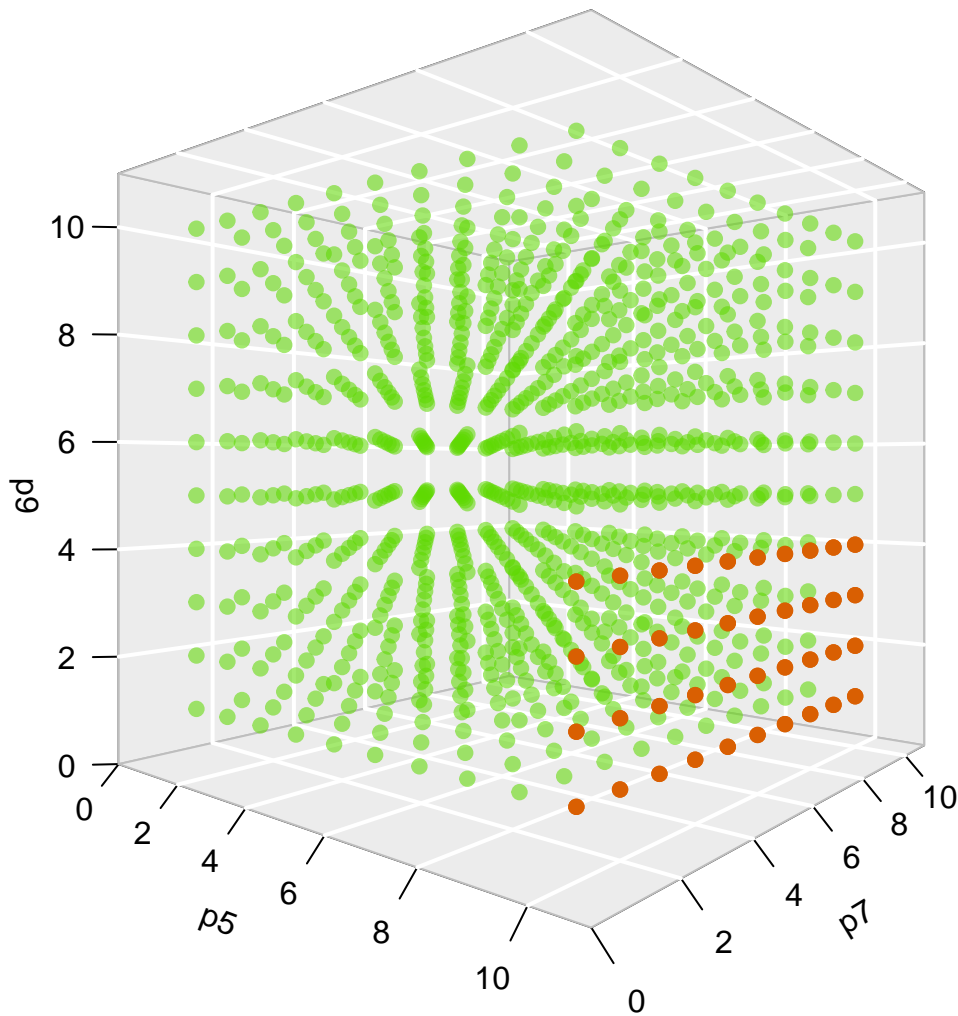

**p35 = 0.35**

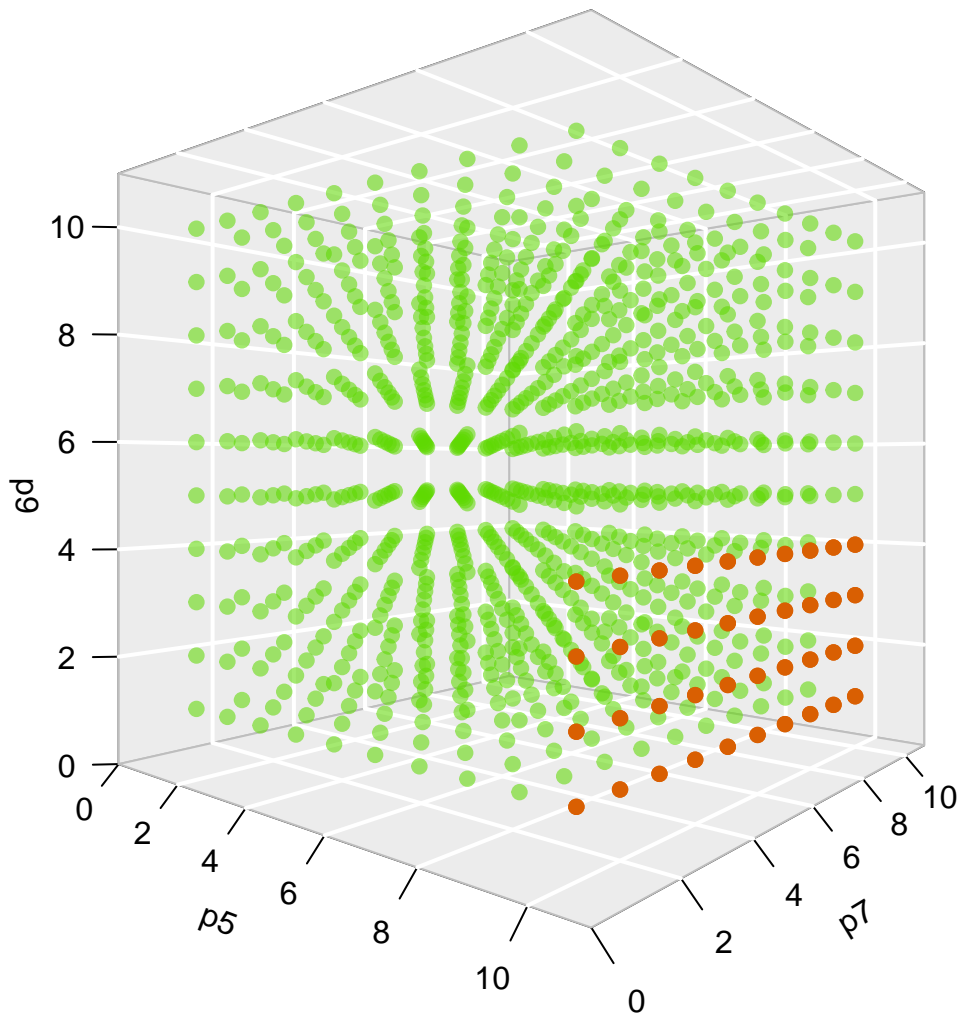

**p35 = 0.42**

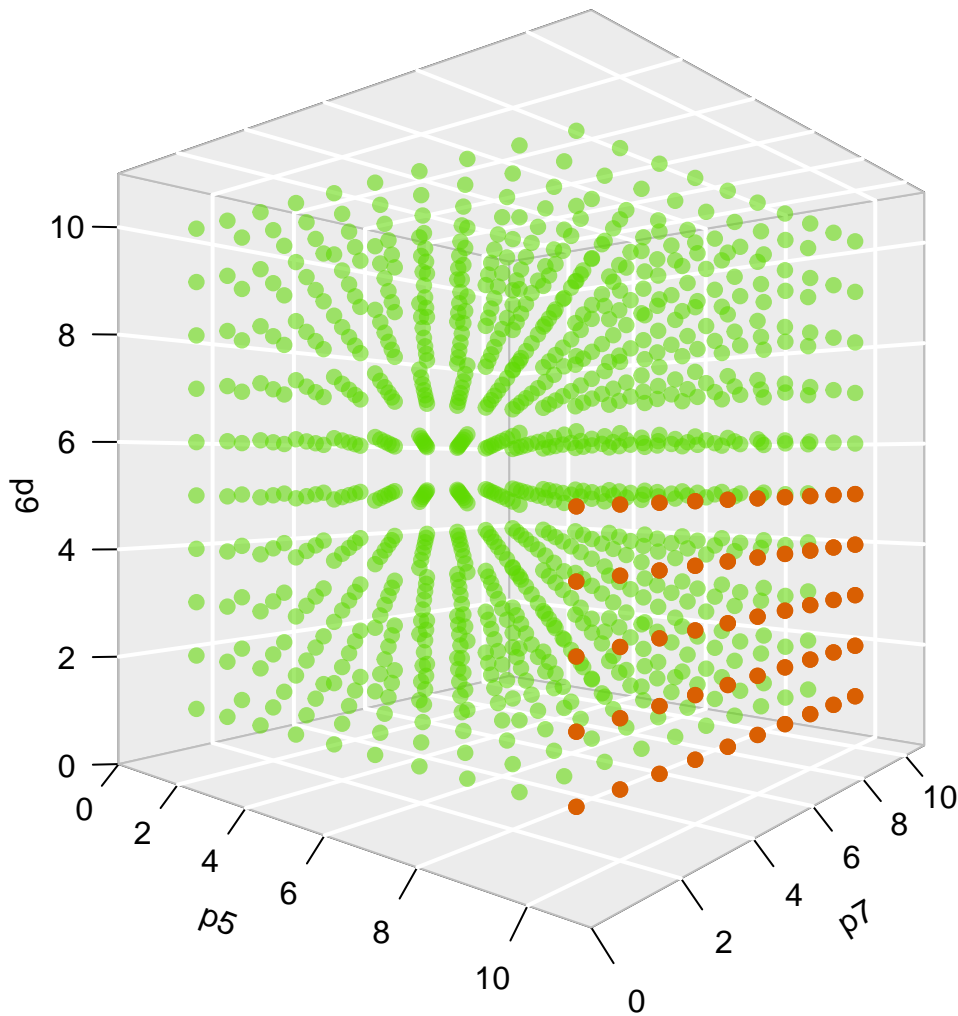

**p35 = 0.56**

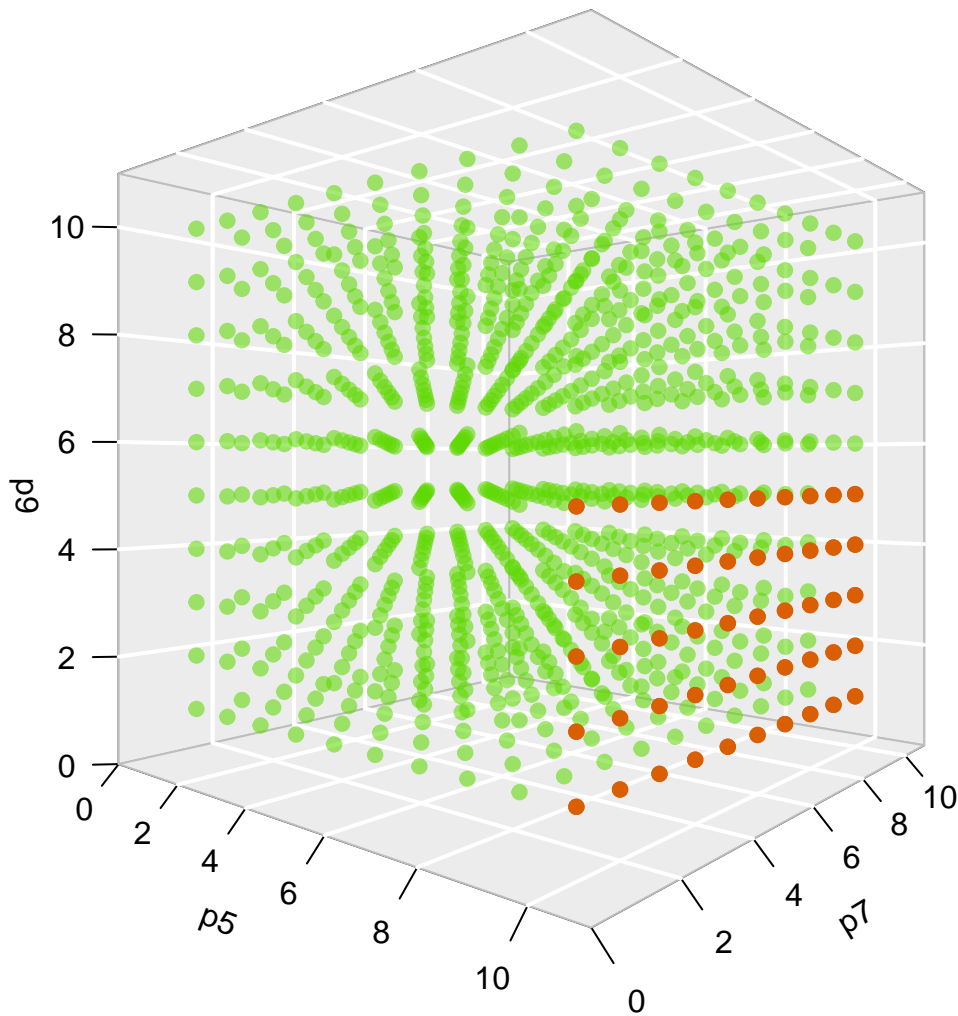

**p35 = 0.63**

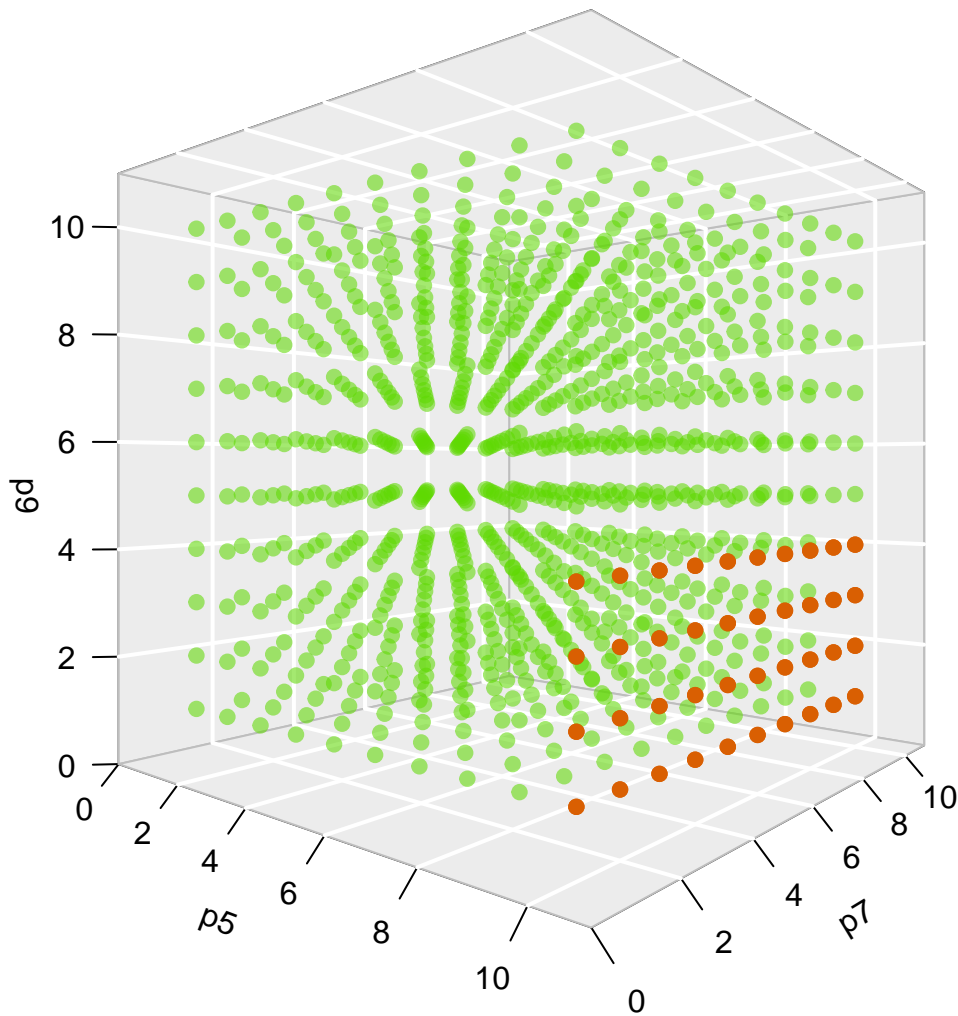

**p35 = 0.85**

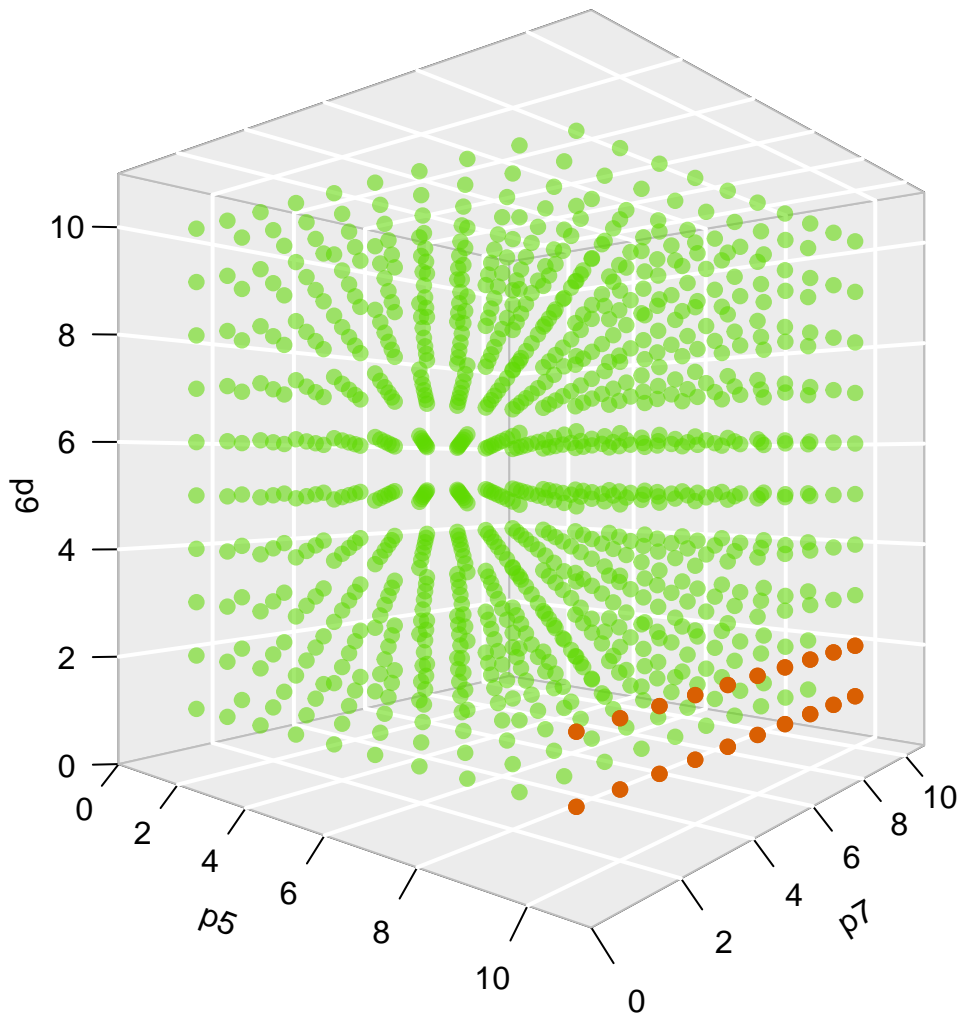

$p_{35} = 1$

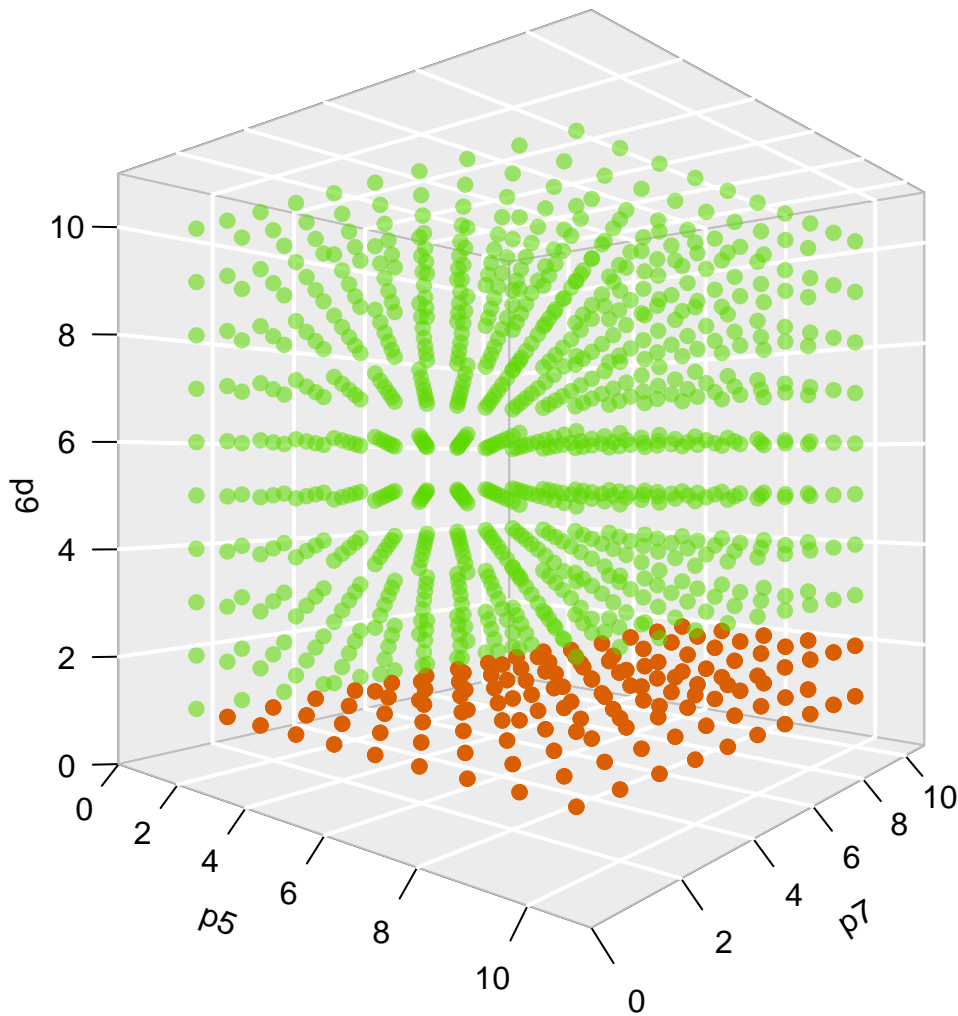

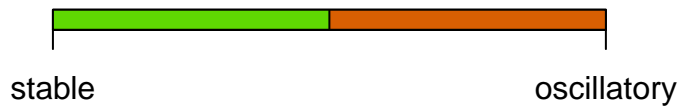

Supplement: Supplementary file 1 [file ijms-24-04806-s001.zip › SF3_Original_oscillation model_manual fitted_3Hills_p35_3d.pdf]

**p35 = 0**

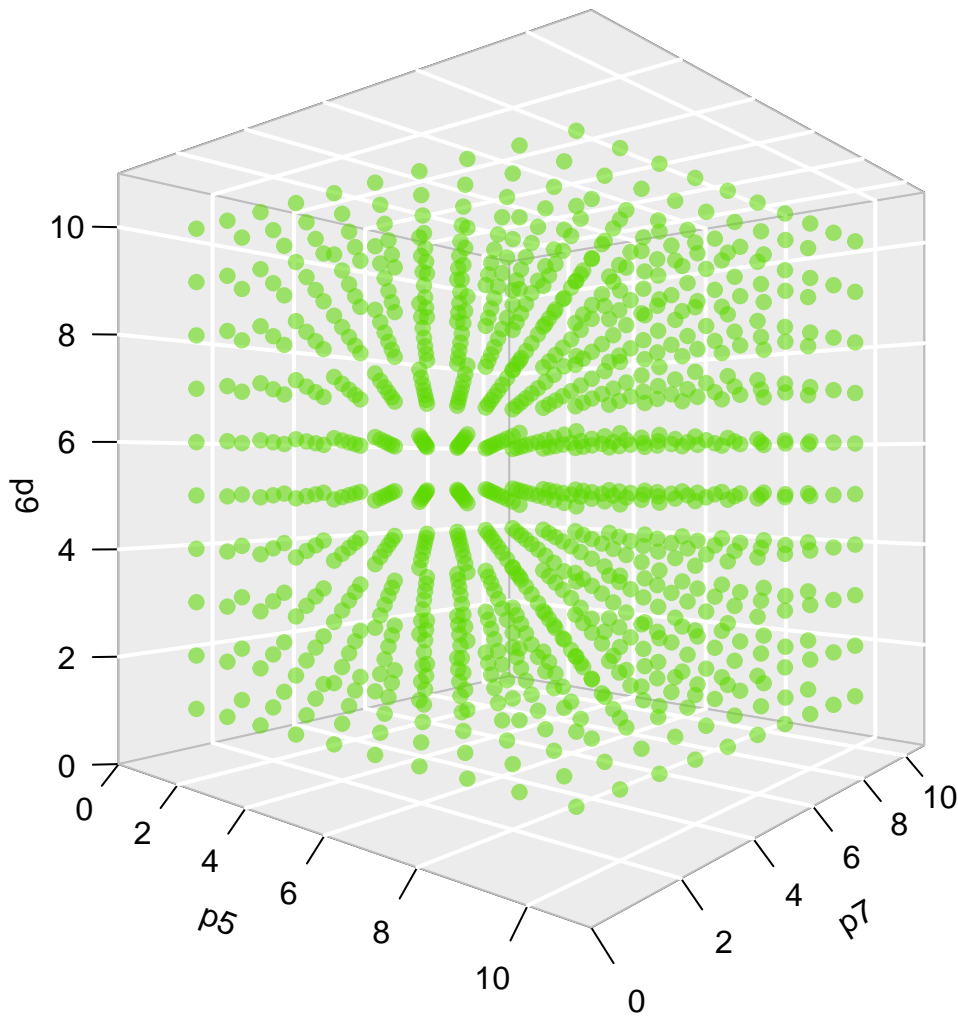

**p35 = 0.07**

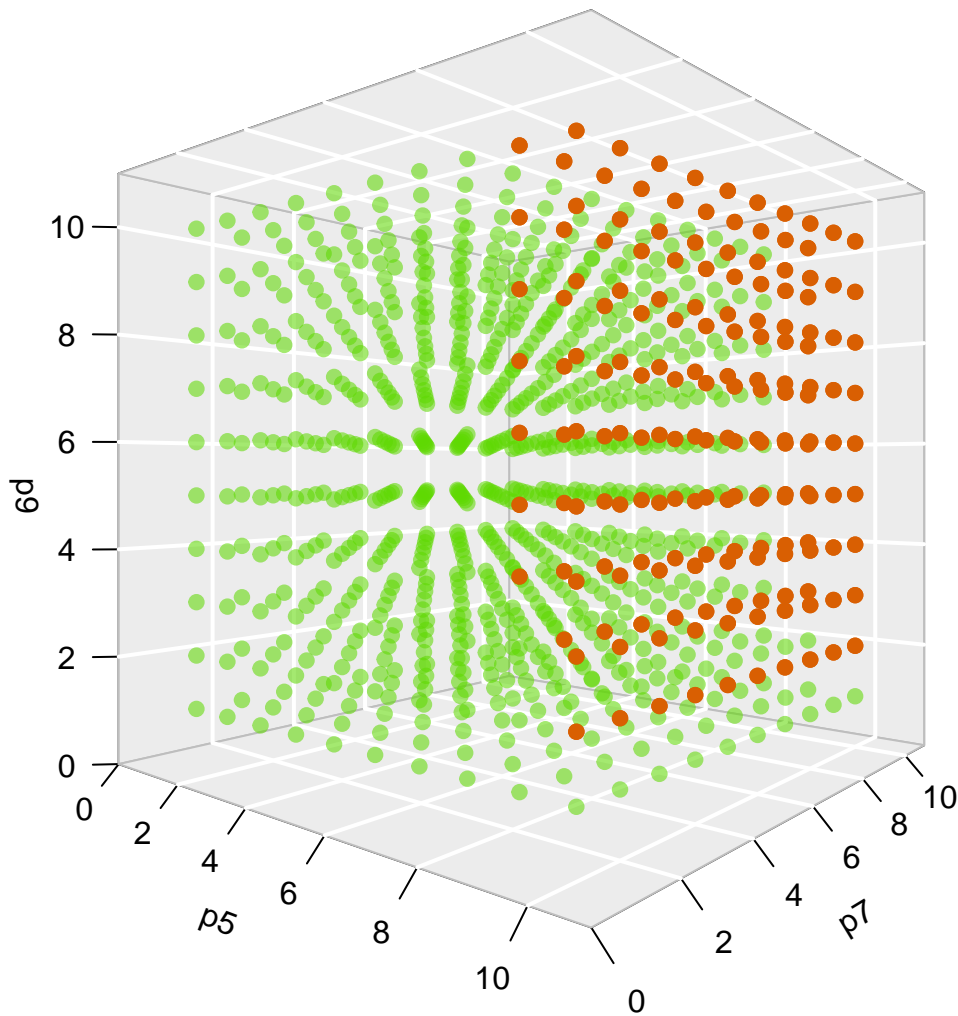

**p35 = 0.14**

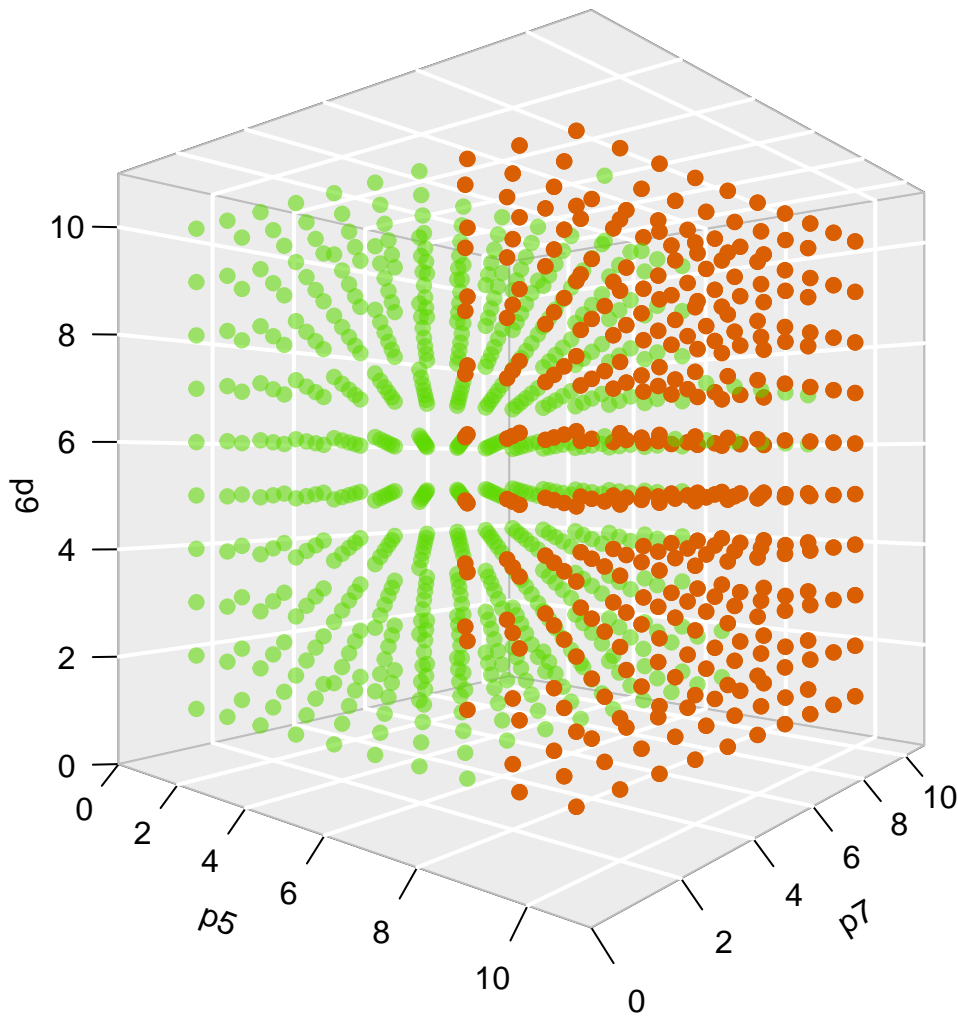

**p35 = 0.21**

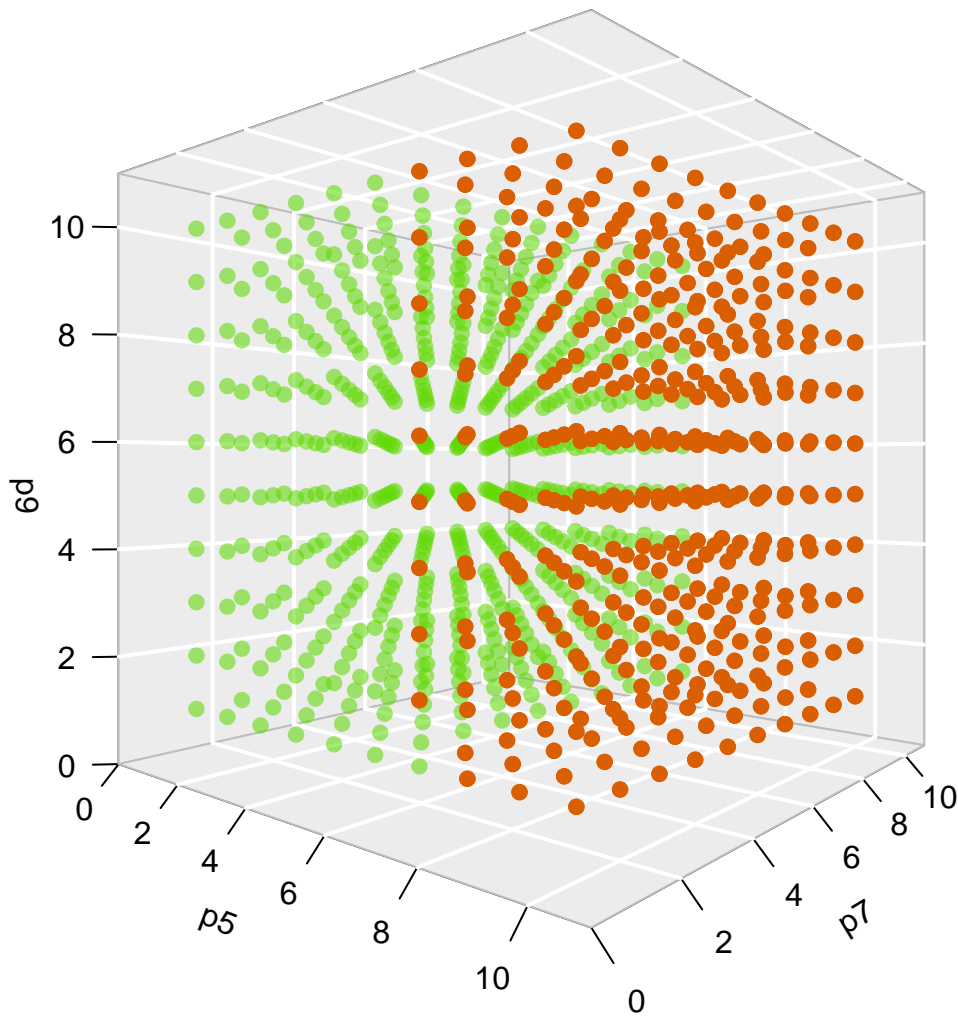

**p35 = 0.28**

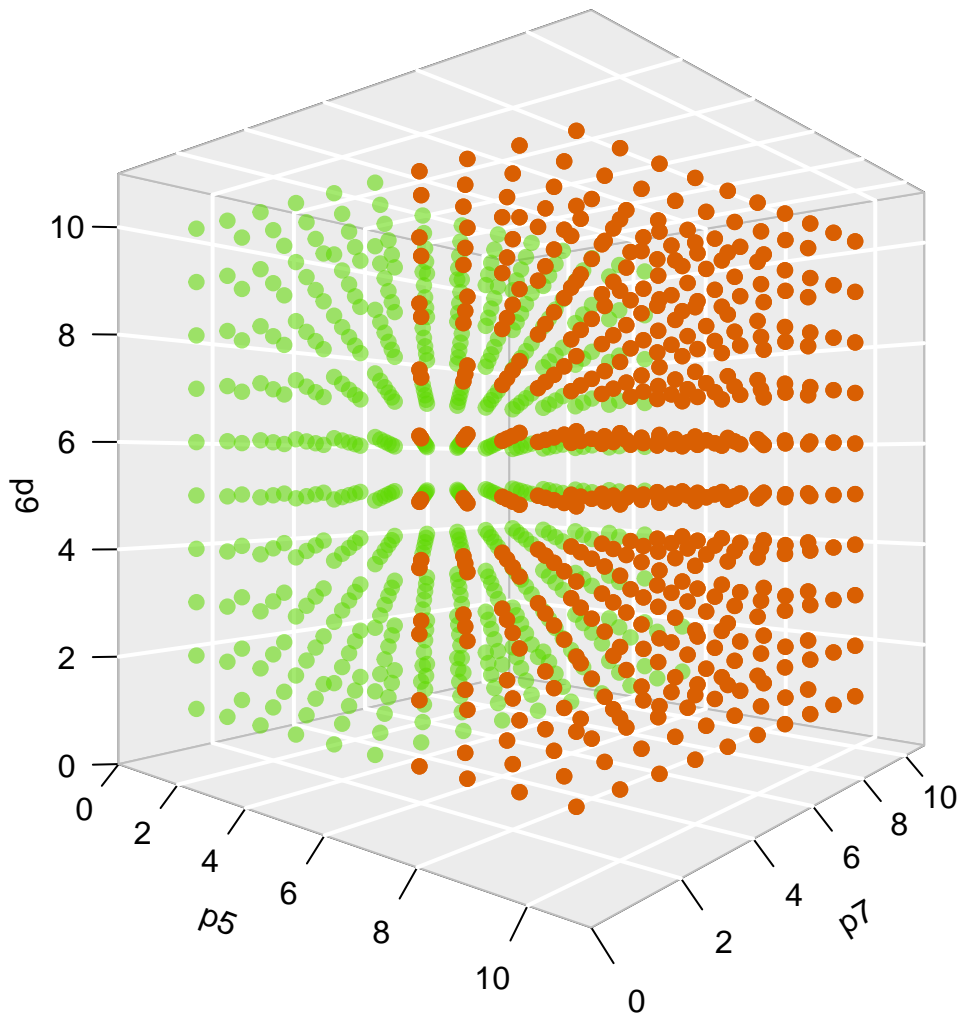

**p35 = 0.35**

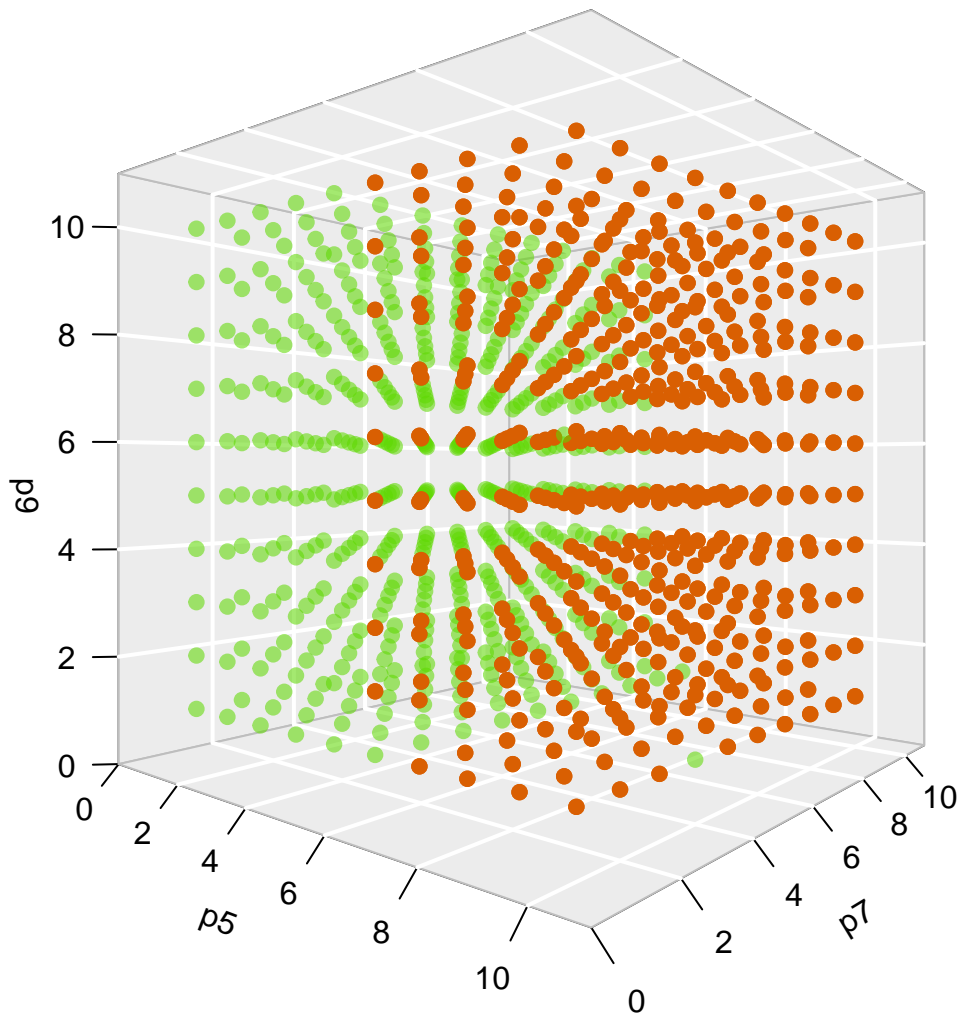

**p35 = 0.42**

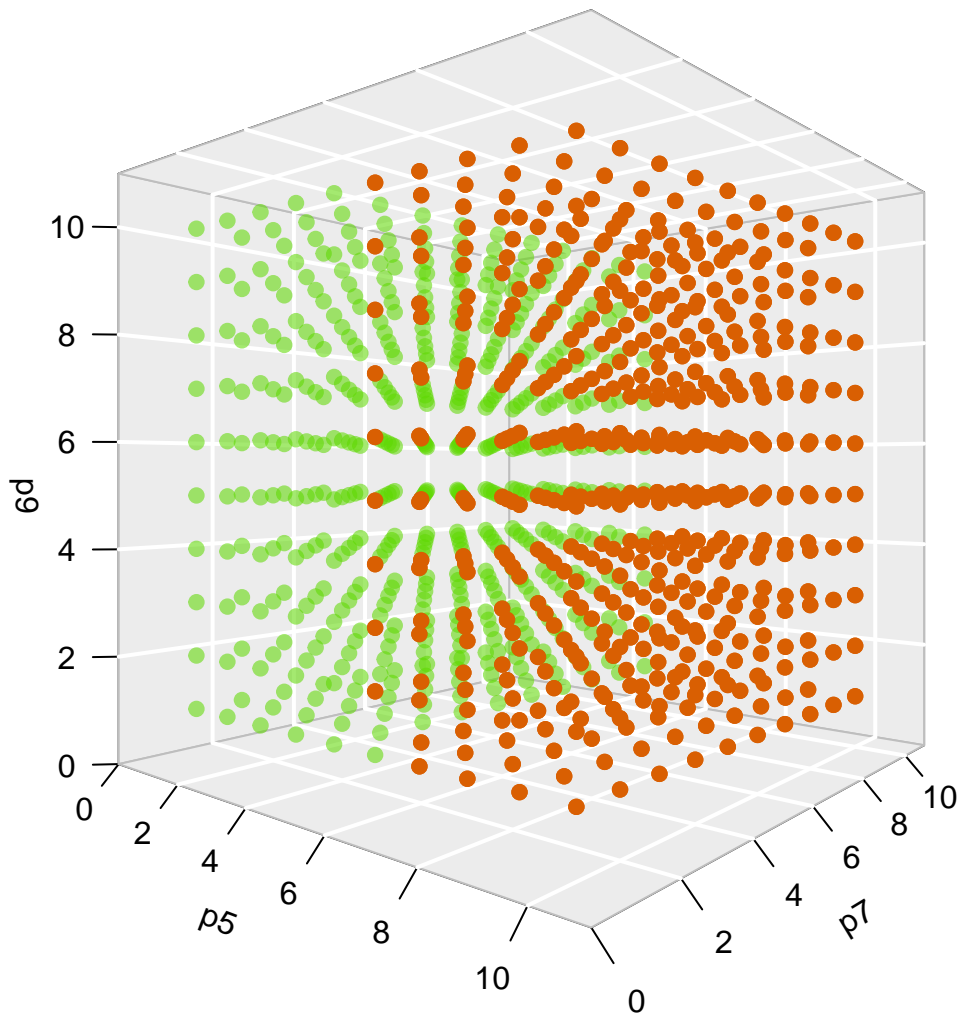

**p35 = 0.56**

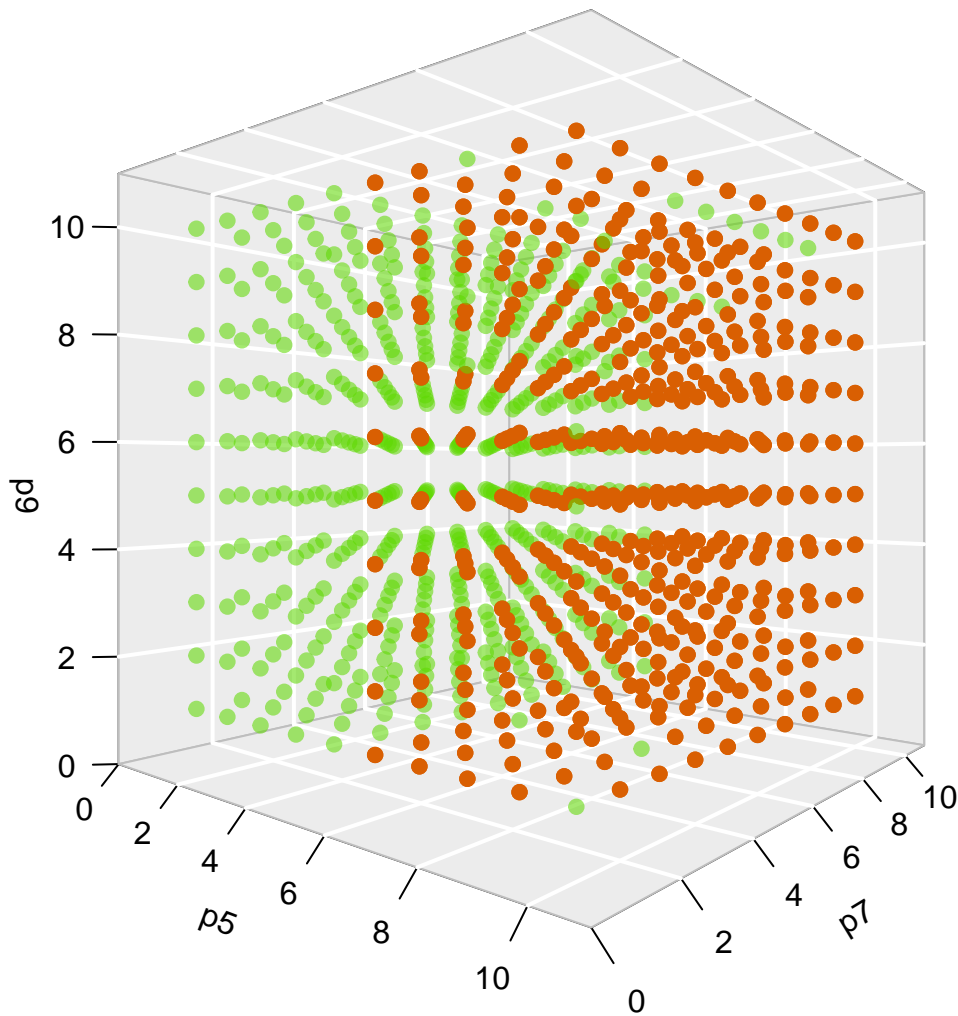

**p35 = 0.63**

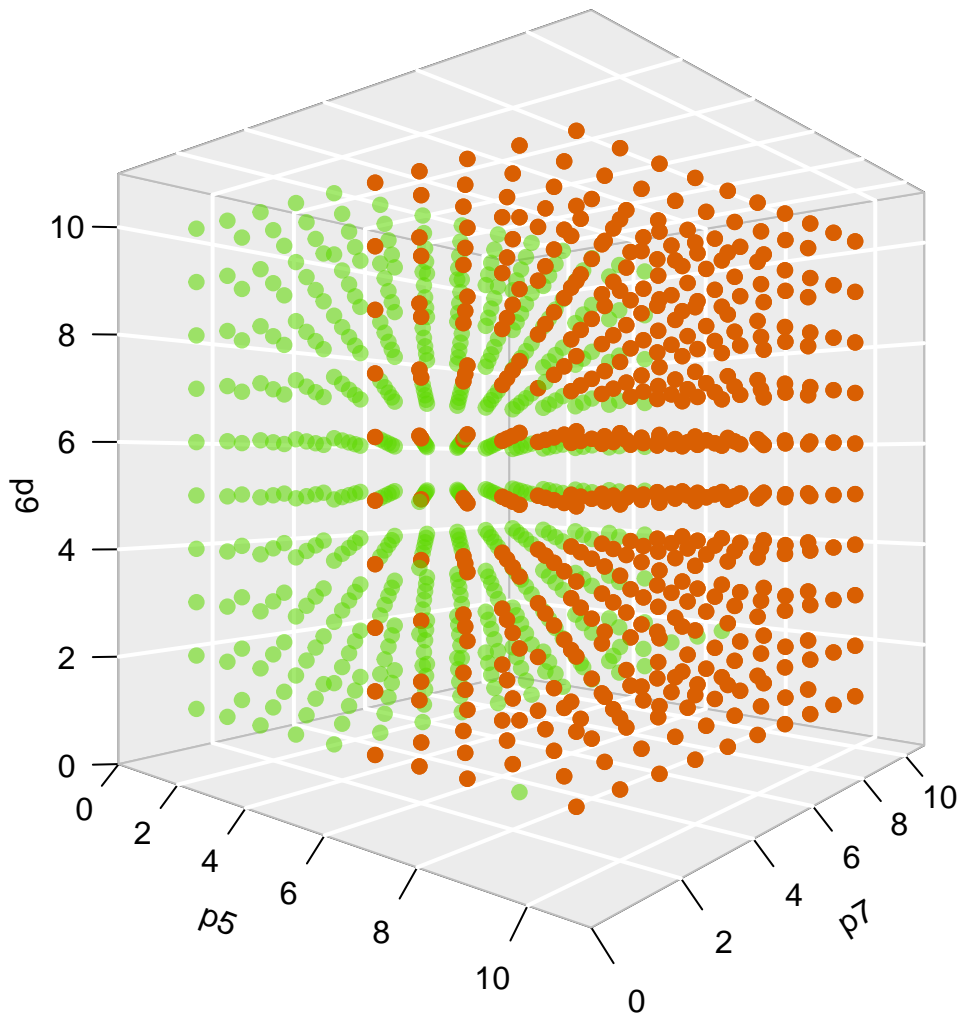

**p35 = 0.85**

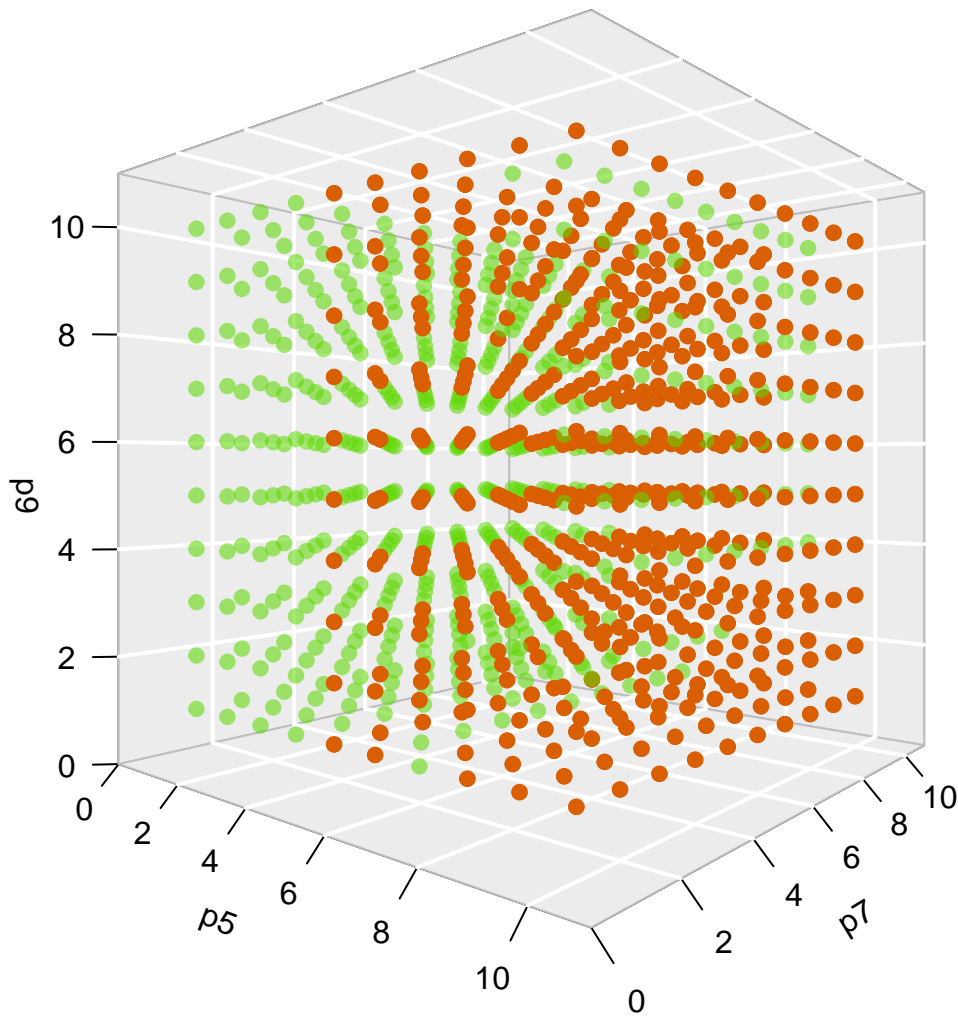

$p_{35} = 1$

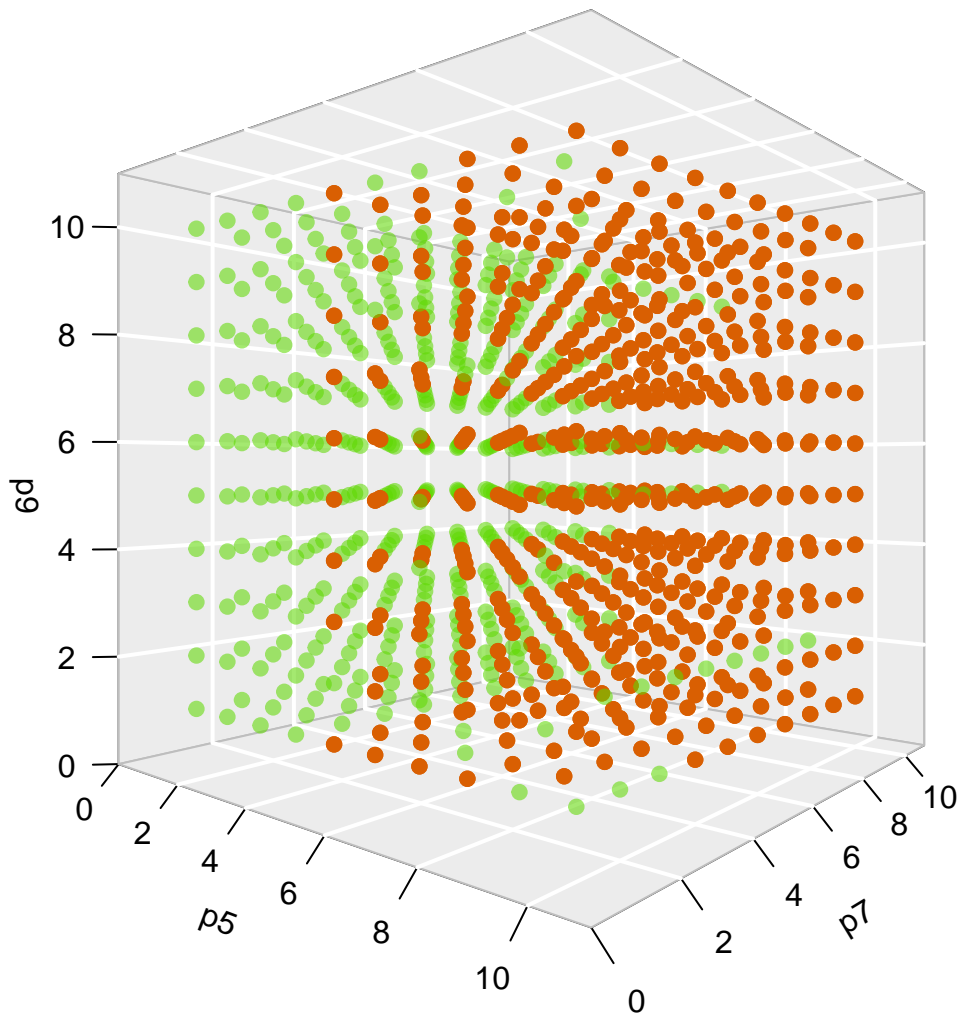

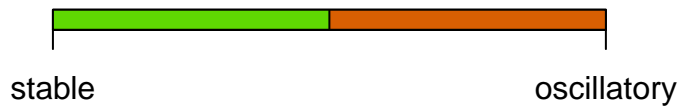

Supplement: Supplementary file 1 [file ijms-24-04806-s001.zip › SF4_Optimized model_Deep_3Hills_p35_3d.pdf]
